# Sphingosine-1-phosphate receptor 3 in the medial prefrontal cortex promotes stress resilience by reducing inflammatory processes

**DOI:** 10.1038/s41467-019-10904-8

**Published:** 2019-07-17

**Authors:** Brian F. Corbett, Sandra Luz, Jay Arner, Jiah Pearson-Leary, Abhishek Sengupta, Deanne Taylor, Philip Gehrman, Richard Ross, Seema Bhatnagar

**Affiliations:** 1 Center for Stress Neurobiology, Children’s Hospital of Philadelphia, Abramson Research Center, 3615 Civic Center Blvd, Philadelphia, PA 19104 USA; 2 Perelman School of Medicine, University of Pennsylvania, Philadelphia, PA 19104 USA; 3 Corporal Michael J. Crescenz Veterans Affairs Medical Center, 3900 Woodland Avenue, Philadelphia, PA 19104 USA

**Keywords:** Stress and resilience, Post-traumatic stress disorder

## Abstract

Stress can promote the development of psychiatric disorders, though some individuals are more vulnerable to stress compared to others who are more resilient. Here we show that the sphingosine-1-phosphate receptor 3 (S1PR3) in the medial prefrontal cortex (mPFC) of rats regulates resilience to chronic social defeat stress. S1PR3 expression is elevated in the mPFC of resilient compared to vulnerable and control rats. Virally-mediated over-expression of S1PR3 in the mPFC produces a resilient phenotype whereas its knock-down produces a vulnerable phenotype, characterized by increased anxiety- and depressive-like behaviors, and these effects are mediated by TNFα. Furthermore, we show that S1PR3 mRNA in blood is reduced in veterans with PTSD compared to combat-exposed control subjects and its expression negatively correlates with symptom severity. Together, these data identify S1PR3 as a regulator of stress resilience and reveal sphingolipid receptors as important substrates of relevance to stress-related psychiatric disorders.

## Abstract (web summary)

S1PR3 is a G protein coupled receptor, that has a role in inflammation. Here the authors show that in the CNS, S1PR3 may contribute to resilience to stressful experiences; resilient rodents show elevated S1pr3, and knockdown results in greater susceptibility to stress.

## Introduction

Chronic or repeated exposure to stress in the form of major life events such as bereavement, prolonged conflict, or low socioeconomic status can increase incidence of depression, generalized anxiety, and post-traumatic stress disorder (PTSD) or exacerbate symptoms in individuals with these disorders^1–3^. However, stress produces these effects only in a vulnerable sub-population of individuals while others remain resilient to the effects of stress^4^. Current treatment strategies are not effective in substantial proportions of individuals affected with stress-related psychiatric disorders^5^ and highlight the need to identify novel substrates that promote resilience to the effects of stress. In studies with adult male rats, we used a paradigm in which a subpopulation of rats exhibits passive coping with rapid onset of submissive defeat postures during repeated daily exposure to an aggressive conspecific. These rats display increases in anxiety-like and pro-depressive behaviors^6–8^ indicating vulnerability (VUL) to the effects of social defeat. The other subpopulation of rats exhibits proactive coping with longer onset to defeat and no changes in anxiety- and depressive-like behaviors compared to non-defeated rats (ND), indicating their resilience (RES) to the effects of repeated social defeat^6–10^. This paradigm is consistent with the human literature in which passive and active coping strategies are important predictors of health outcomes^11^. Therefore, we predicted that segregation of defeated animals by coping strategy would allow us to identify neural mechanisms that promote resilience or vulnerability to stress.

Sphingosine-1-phosphate receptor 3 is a G protein-coupled receptor that, upon binding to its ubiquitous ligand sphingosine-1-phosphate (S1P), plays a critical role in regulating multiple cellular processes including inflammation, migration, angiogenesis, differentiation, and proliferation in peripheral tissue^12–19^. Little is known about the function of S1PR3 in the brain, although a role for S1PR3 in spatial working memory and excitability of hippocampal neurons in rodents has been reported^20^. In this study, we identify S1PR3 in the mPFC as a key mediator of resilience to the adverse effects of stress in rats. S1PR3 mRNA and protein are increased in the mPFC of RES rats. This increase is causatively related to stress resilience as S1PR3 knock-down in the mPFC promotes vulnerability to stress and S1PR3 over-expression promotes resilience in defeated rats. S1PR3 knock-down in the mPFC exacerbates defeat-induced increases in TNFα. Attenuation of stress-induced increases in TNFα in the mPFC is an important mechanism by which S1PR3 promotes resilience as blocking TNFα signaling rescues the vulnerable phenotype caused by S1PR3 knock-down. We provide evidence that stress increases S1PR3 expression in RES, but not VUL, rats through glucocorticoid receptor (GR)-mediated mechanisms. Finally, we provide evidence that blood *S1PR3* mRNA expression is reduced in PTSD patients compared to combat-exposed veterans without PTSD and blood *S1PR3* mRNA is negatively correlated with PTSD symptom severity. Together, our findings demonstrate that S1PR3 in the mPFC promotes resilience to stress and is a novel substrate of relevance to stress-related psychiatric disorders.

## Results

### S1PR3 expression is increased in the mPFC of resilient rats

**Fig. 1:**
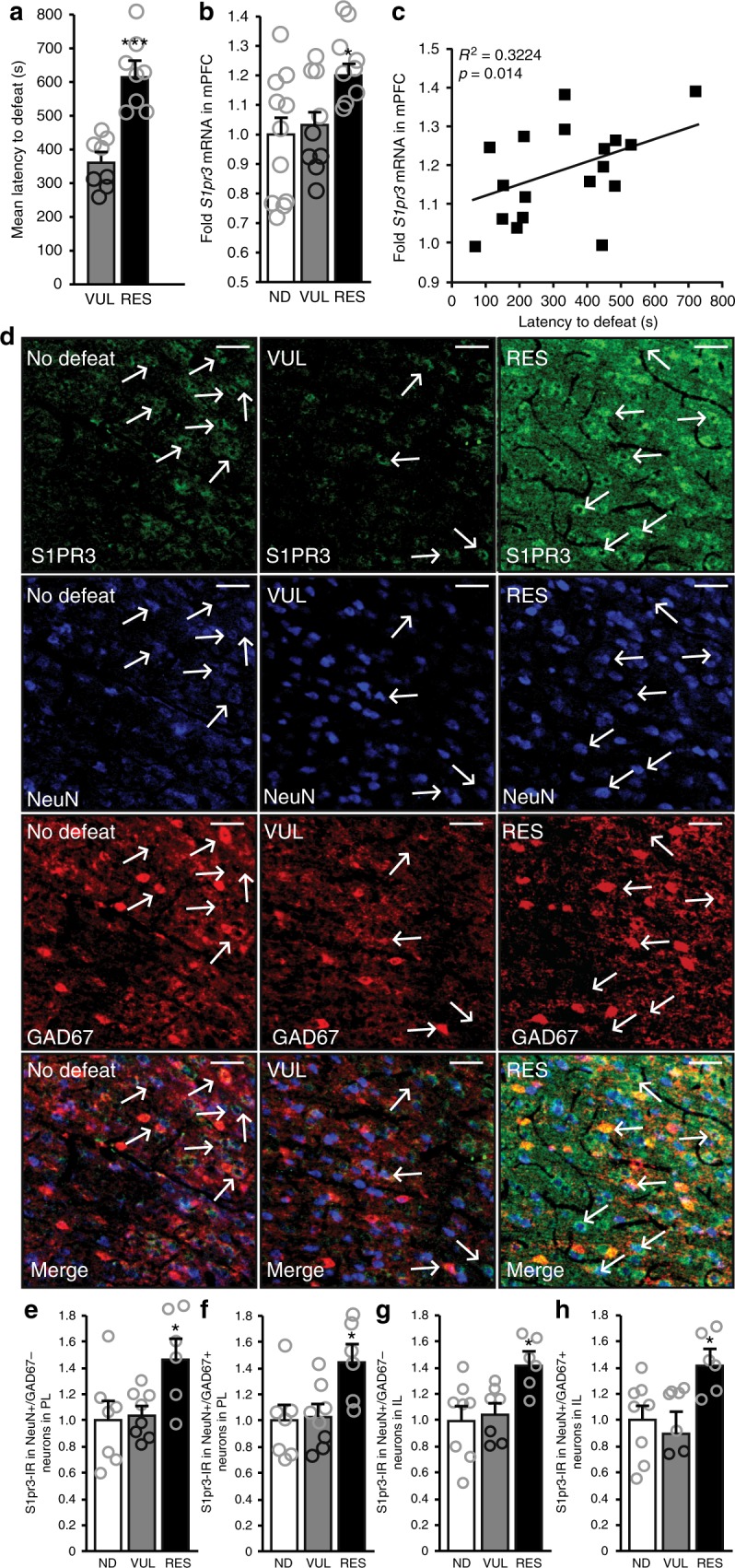
S1PR3 is increased in the mPFC of resilient rats. **a** Mean defeat latencies averaged over 7 days in VUL and RES rats (*n* = 8/group, Student’s *t*-test). **b** Fold mPFC *S1pr3* mRNA (relative to ND controls) in ND (*n* = 11), VUL (*n* = 9), and RES (*n* = 9) rats. **c** Correlation between mean defeat latency and mPFC *S1pr3* mRNA expression in VUL and RES rats. **d** Images (captured at 20×) and quantification of S1PR3 protein immunoreactivity (IR) indicating increased expression in RES rats (*n* = 6) compared to ND (*n* = 7) and VUL (*n* = 8) rats in **e** GAD67− neurons in PL **f** GAD67+ neurons in PL, **g** GAD67− neurons in IL, and **h** GAD67+ neurons in IL. White lines in the top right corner of each panel represent 50 µm. Bars represent means + SEM. For **a**, *p < 0.05 using Student’s *t*-test. For **b**, **e**–**h**, ***p < 0.001, *p < 0.05 in RES compared ND and VUL rats as calculated by Tukey’s post-hoc test following one-way ANOVA. Arrows indicate S1PR3-IR neurons that are + or − for GAD67

To identify novel targets associated with resilience or vulnerability, a PCR array was used to screen for novel genes in the medial prefrontal cortex (mPFC), a region uniquely situated to regulate resilience/vulnerability to stress as it mediates stress-induced changes in affective behavior, the neuroendocrine response, and executive and cognitive functions that are adversely impacted by stress^21–24^. Indeed, coherence of mPFC network activity with limbic structures has been demonstrated to be a predictor for resilience/vulnerability to stress^25^. Of the 192 genes examined in this array, 10 were differentially expressed between rats characterized as RES and VUL (complete results provided in Supplementary Table 1, defeat latencies presented in Fig. 1a). One of these was the *Sphingosine-1- phosphate receptor 3* (*s1pr3*, also known as *edg3*). Its expression was significantly higher in the RES compared to ND and VUL rats (Fig. 1b) and positively correlated with mean defeat latencies averaged across seven days (Fig. 1c). Identification of S1PR3 was unexpected because little is known about the function of S1P receptors in the brain. The finding of increased *S1pr3* mRNA in the mPFC was specific to this particular S1P receptor since *S1pr1* and *S1pr2* mRNA in the mPFC was not different among ND control, VUL, and RES rats (Supplementary Fig. 1a, b). Increased *S1pr3* expression occurred without a compensatory change in mPFC sphingosine-1-phosphate (S1P; Supplementary Fig. 1c), suggesting that activity of the S1PR3 signaling pathway is increased. S1PR3 is expressed in neurons^20^, astrocytes^26,27^, and microglia^27^, but a fuller understanding of the neuronal subtypes expressing S1PR3 in the rat mPFC is lacking. We triple-labeled mPFC neurons for S1PR3, the neuronal marker NeuN, and the inhibitory neuronal marker GAD67. Compared to ND control and VUL rats, RES rats displayed increased S1PR3-immunoreactivity (IR) in both excitatory (NeuN+/GAD67−) and inhibitory (NeuN+/GAD67+) neurons in the prelimbic (PL) and infralimbic (IL) cortices of the mPFC (Fig. 1d–h). Relative increases in RES compared to ND or VUL rats were greater in some layers (Supplementary Fig 1d–h). In parallel, we performed immunohistochemistry comparing S1PR3 in GFAP-expressing (astrocytes) and IBA1-expressing cells (microglia and monocytes). S1PR3 expression in astrocytes and microglia was low compared to neuronal expression, with no differences observed among ND, VUL, and RES rats (Supplementary Fig 1i–k). Together, these data provided evidence that S1PR3 expression is increased in both inhibitory and excitatory neurons and in multiple layers of the mPFC in rats resilient to the effects of repeated social defeat. Based on these findings, we hypothesized that elevated S1PR3 in the mPFC mediates resilience to stress.

### S1PR3 knock-down in the mPFC promotes stress vulnerability

**Fig. 2:**
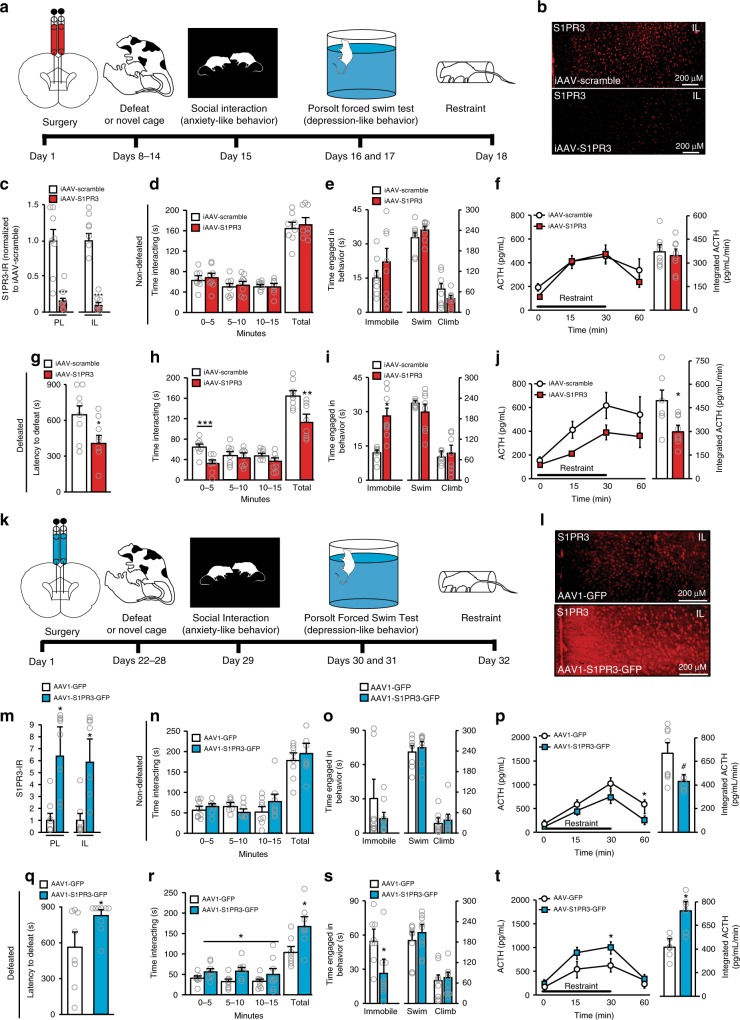
S1PR3 in the mPFC is necessary and sufficient to promote stress resilience. **a** Experimental paradigm for assessing behavioral and neuroendocrine phenotypes following S1PR3 knock-down. **b** Image and **c** quantification of S1PR3-IR cells in the PL and IL of iAAV-scramble (*n* = 8) and iAAV-S1PR3 (*n* = 8) rats at the end of the testing paradigm, 18 days following injection. In non-defeated rats, **d** time interacting with stimulus rat in the social interaction paradigm (*n* = 8/group), **e** time engaged in immobile, swim, or climb behaviors in the 5 min test phase of the Porsolt Forced Swim Test (*n* = 8/group), and **f** plasma ACTH concentrations in response to restraint; integrated plasma ACTH production over 60 min (*n* = 7/group). In defeated rats, **g** mean defeat latencies over 7 days of resident-intruder paradigm (*n* = 8/group), **h** time interacting with the stimulus rat (*n* = 8/group), **i** time engaged in behavior during the test phase of the Porsolt Forced Swim Test (*n* = 8/group), and **j** plasma ACTH concentrations in response to restraint; integrated plasma ACTH (iAAV-scramble *n* = 6, iAAV-S1PR3 *n* = 8). **k** Experimental paradigm for assessing behavioral and neuroendocrine phenotypes following S1PR3 over-expression. **l** Image and **m** quantification of S1PR3-IR (percent area over threshold relative to controls) in the PL and IL (*n* = 8/group). In non-defeated rats, **n** time interacting with the stimulus rat (*n* = 8/group), **o** time engaged in behavior in the 5 min test phase of the Porsolt Forced Swim Test (*n* = 8/group), and **p** plasma ACTH concentrations in response to restraint ; integrated plasma ACTH production over 60 min (AAV1-GFP n = 8, AAV1-S1PR3-GFP *n* = 6). In defeated rats, **q** mean defeat latencies (AAV1-GFP *n* = 8, AAV1-S1PR3-GFP *n* = 9), **r** time interacting with the stimulus rat (AAV1-GFP *n* = 8, AAV1-S1PR3-GFP *n* = 9), **s** time engaged in behavior during the test phase of the Porsolt Forced Swim Test (AAV1-GFP *n* = 8, AAV1-S1PR3-GFP *n* = 9), and **t** plasma ACTH concentrations during restraint paradigm; integrated blood plasma ACTH production over 60 min (*n* = 6/group). Bars represent means + SEM. *p < 0.05, **p < 0.01, ***p < 0.001, #*p* < 0.07, Student’s *t*-test. Horizontal bars, asterisk (*) represent Bonferonni post-hoc differences following repeated measures two-way ANOVA in (**h**, **j**, **p**, **r**, and **t**)

To test this hypothesis, we investigated whether elevated S1PR3 in the mPFC was necessary and/or sufficient to promote the behavioral and neuroendocrine phenotypes associated with resilience (Fig. 2a). We used viral tools to knock-down or over-express S1PR3 in the mPFC because pharmacological tools specifically targeting S1PR3 are not established in vivo. Bilateral injections of iAAV-S1PR3 in the mPFC knocked down S1PR3, reducing the number of S1PR3-immunoreactive (IR) cells by >70% (Fig. 2b, c). Reductions in S1PR3-IR were observed as early as 7 days following injection (Supplementary Fig. 2a, b), suggesting that reductions in S1PR3 were present at the time of first defeat in iAAV-S1PR3-injected rats. Knock-down of S1PR3 had no effects in ND rats (Fig. 2d–f). However, S1PR3 knock-down in the mPFC of socially defeated rats promoted a more vulnerable phenotype relative to control iAAV-scramble rats. iAAV-S1PR3 rats displayed passive coping during daily defeat (reduced mean defeat latencies), increased anxiety-like behavior (decreased time interacting with the stimulus rat in the social interaction test), pro-depressive behavior (increased immobility in the Porsolt forced swim test [FST]) and impaired adrenocorticotropic hormone (ACTH) responses to novel restraint challenge (Fig. 2g–j). Thus, reducing S1PR3 expression in the mPFC shifted defeated rats towards a vulnerable phenotype.

### S1PR3 over-expression in the mPFC promotes stress resilience

To determine whether the converse, overexpression of S1PR3 in the mPFC, promoted a resilient phenotype (paradigm illustrated in Fig. 2k), we bilaterally administered either AAV1-GFP (control virus) or AAV1-S1PR3-GFP, which non-specifically overexpresses GFP or overexpresses S1PR3 and GFP, respectively (overexpression confirmed in Fig. 2l, m and Supplementary Fig. 2c). S1PR3 overexpression did not alter behavioral phenotypes in ND rats (Fig. 2n, o), though it modestly reduced ACTH response to the novel stress of restraint (Fig. 2p). This may indicate that, with sufficiently high expression, S1PR3s are important in limiting HPA responses to acute stressors. Importantly, in defeated rats, S1PR3 overexpression promoted a more resilient phenotype relative to AAV1-GFP control rats as AAV1-S1PR3-GFP-treated rats displayed increased mean defeat latencies, increased time interacting with the stimulus rat in the social interaction test, reduced immobility in the FST, and facilitated ACTH production during restraint (Fig. 2q–t). Together, these experiments provided strong evidence that S1PR3s in the mPFC are both necessary and sufficient to promote resilience to the adverse effects of stress.

### S1PR3 regulates stress-mediated effects on mPFC network activity

Behavior is regulated by the activity of cellular networks working in concert. To specifically determine the role of S1PR3s in regulating mPFC network activity in stressed rats, we examined local field potentials (LFPs) in the mPFC. Coping strategies become differentiated into passive and active phenotypes by the 5th defeat exposure. Therefore, LFP recordings from rats injected with iAAV-scramble or iAAV-S1PR3 in the mPFC were examined prior to, and immediately after, defeat on day 1 and day 5 (recordings during defeat were technically unfeasible; Supplementary Fig. 2). Following the first day of defeat, iAAV-S1PR3 rats displayed increased power spectral densities in the delta (1.5–4 Hz) range but these increases were not observed in control scramble rats until after the fifth defeat. Thus, knock-down of S1PR3 produced network activity in the mPFC in response to a single defeat that is otherwise not significantly exhibited until repeated defeat exposure. Recent work shows that, compared to resilient mice, stress-vulnerable mice exhibit increased delta oscillations originating from the PFC and nucleus accumbens that influence activity in the basolateral nucleus of the amygdala^28^ and are associated with fear-induced freezing^29^. These findings suggest that the network activity regulated by S1PR3 in the mPFC influences activity in the amygdala and amygdala-regulated behaviors. Similar to the findings with power in the delta range, iAAV-S1PR3 rats displayed a significant decrease in high theta (6–8 Hz) power spectral densities following a single defeat whereas iAAV-scramble rats only displayed a significant change in network activity following five defeats. Thus, again knock-down of S1PR3 advanced the impact of stress on mPFC oscillatory network activity. Additionally, compared to iAAV-scramble controls, iAAV-S1PR3 rats displayed increased gamma oscillations (32–40 Hz) at baseline (pre-defeat day 1), post-defeat day 1, and post-defeat day 5 time points compared to scramble controls (Supplementary Fig. 2d, e). Gamma oscillations in the mPFC are associated with enhanced attentional capacity^30,^ suggesting that the vulnerable phenotype of iAAV-S1PR3 rats may be related to exaggerated attention to stressful stimuli. Together, these data provide evidence that S1PR3s normally act to delay the impact of repeated social defeat on the network activity of the mPFC.

### S1PR3 regulates neuronal activity in the extended mPFC network

We investigated whether S1PR3 over-expression in the mPFC altered neuronal activity in stress-related brain regions receiving input from the mPFC. AAV1-GFP and AAV1-S1PR3 rats underwent seven days of social defeat and were sacrificed 60 min following the onset of a 30 min restraint. The bed nucleus of the stria terminalis (BNST) receives input from the mPFC^31^ and the posterior (p)BNST inhibits the paraventricular nucleus of the hypothalamus (PVN)^32,33^, the hypothalamic arm of the HPA axis^34^. Consistent with their facilitated ACTH response to acute restraint, AAV1-S1PR3 rats exhibited fewer c-Fos-IR neurons in the pBNST (Supplementary Fig. 3a, b) and a greater number of c-Fos-IR neurons in the PVN (Supplementary Fig. 3c, d) compared to control rats at 60 min following the onset of a 30 min restraint. Additionally, we assessed c-Fos expression in the amygdala, in which activity is generally inhibited by the mPFC and which regulates fear behavior^35^. Compared to controls, AAV1-S1PR3 rats displayed fewer c-Fos-IR neurons in the basolateral nucleus of the amygdala (BLA, Supplementary Fig. 3e, f), but not the central nucleus of the amygdala (CeA, Supplementary Fig. 3g, h). Together, these data suggest that S1PR3s in the mPFC influence neuronal activity in downstream targets, including increasing activity in those structures associated with facilitated ACTH production and reducing activity in a structure related to fear and emotional arousal.

### S1PR3 knock-down exacerbates stress-induced inflammation

**Fig. 3:**
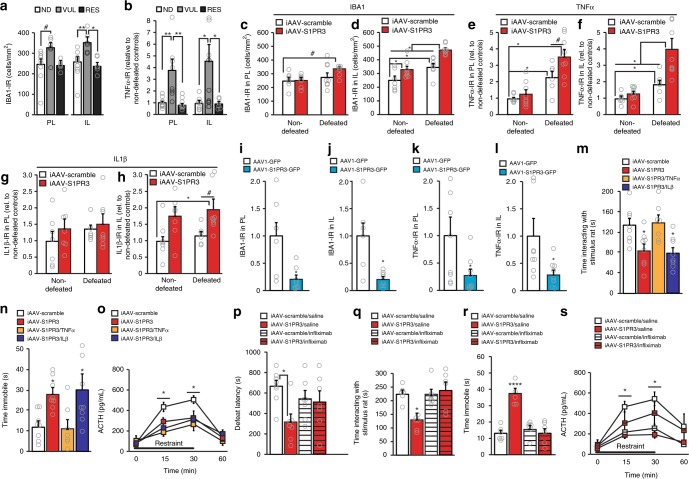
S1PR3 promotes stress resilience by attenuating stress-induced inflammatory processes in the mPFC. **a** IBA1-IR cell density in PL and IL of ND (*n* = 10), VUL (*n* = 6), and RES (*n* = 6) rats. **b** TNFα-IR (percent area above threshold) in PL and IL of ND (*n* = 8), VUL (*n* = 8), and RES (*n* = 7) rats. IBA1-IR cell density in **c** PL and **d** IL of non-defeated and defeated iAAV-scramble and iAAV-S1PR3 rats (non-defeated iAAV-scramble *n* = 7, others *n* = 8/group). TNFα-IR in **e** PL and **f** IL. IL1β-IR (percent area above threshold) in **g** PL and **h** IL. IBA1-IR (percent area above threshold) in **i** PL and **j** IL of AAV1-GFP (*n* = 6) and AAV1-S1PR3 (*n* = 9) rats. TNFα-IR (percent area above threshold) in **k** PL and **l** IL of AAV1-GFP (*n* = 6) and AAV1-S1PR3 (*n* = 9) rats. **m** iAAV-S1PR3 rats, but not iAAV-S1PR3/TNFα rats, displayed decreased time interacting with the stimulus rat compared to iAAV-scramble rats (iAAV-S1PR3/TNFα *n* = 7, others *n* = 8/group). **n** iAAV-S1PR3 rats, but not iAAV-S1PR3/TNFα rats, displayed increased time immobile in the test phase of the Porsolt Forced Swim Test compared to iAAV-scramble rats (iAAV-S1PR3/TNFα *n* = 7, others *n* = 8/group). **o** Plasma ACTH concentrations in response to restraint (iAAV-S1PR3/TNFα *n* = 7, others *n* = 8/group). **p** Mean latency to defeat was reduced in saline-treated, but not infliximab-treated, iAAV-S1PR3 rats compared to saline-treated iAAV-scramble rats (*n* = 7/group). **q** Time interacting with the stimulus rat was reduced in saline-treated, but not infliximab-treated, iAAV-S1PR3 rats compared to saline-treated iAAV-scramble rats (iAAV-scramble/infliximab *n* = 7, others *n* = 6/group). **r** Time immobile in the Porsolt Forced Swim Test was increased in saline-treated, but not infliximab-treated, iAAV-S1PR3 rats compared to saline-treated iAAV-scramble rats (iAAV-S1PR3/saline *n* = 5, others *n* = 6/group). **s** Plasma ACTH responses to restraint were reduced in iAAV-S1PR3/saline and iAAV-scramble/infliximab rats compared to controls at 15 and 30 min timepoints. Bars represent mean + SEM. *p < 0.05, ***p < 0.001, ****p < 0.0001, #*p* < 0.07. Horizontal bars represent post-hoc differences. Statistical tests: **a**, **b** Tukey/one-way ANOVA; **c**–**h**, **p**–**r** Bonferonni/two-way ANOVA; **i**–**l** Student’s *t*-test; **m**, **n** Holm-Sidak’s/one-way ANOVA; **o**, **s** Bonferonni/repeated measures two-way ANOVA). For **m** and **n**, asterisk (*) represents difference from iAAV-scramble rats. For **p** and **r**, * and **** indicate differences from all other groups

We next investigated the cellular mechanisms through which S1PR3s in the mPFC promote resilience. While their effects on inflammation in the brain are not known, S1PR3s reduce inflammation in peripheral tissue^12,13^. Based on this known role of S1PR3s in the periphery and because some inflammatory markers are increased in the PFC of socially-defeated mice^36^, we hypothesized that S1PR3s promote resilience by attenuating stress-induced inflammatory processes in the mPFC. We first assessed markers of inflammatory processes in the mPFC of ND, RES and VUL rats. We quantified the density of microglia and monocytes, which express ionized calcium-binding adapter molecule 1 (IBA1) and serve as the primary mediators of inflammation in the brain^8,37,38^. In the PL, there was a trend for increased IBA1-IR cell density of VUL rats compared to ND rats following 7 days of social defeat. In the IL, IBA1-IR cell density was significantly increased in VUL rats compared to ND and RES rats (Fig. 3a). Previous studies have demonstrated that repeated social defeat increased the expression of tumor necrosis factor α (TNFα) mRNA in the PFC^36^, an inflammatory cytokine that contributes to anxiety- and depression-like behavior in rodents^39^ and symptoms of depression in humans with elevated inflammatory markers^40^. We observed increased TNFα-IR in the PL and IL of VUL rats compared to ND and RES rats following 7 days of social defeat (Fig. 3b). These results suggested increases in pro-inflammatory processes in the mPFC of VUL rats.

To investigate the role of S1PR3 in inflammatory processes in socially defeated rats, we examined IBA1-IR cell densities in ND or socially defeated rats injected with either iAAV-scramble or iAAV-S1PR3. In the IL sub-region of the mPFC, the density of IBA1-IR cells was higher in defeated rats compared to non-defeated rats with scramble control virus (Fig. 3d and Supplementary Fig. 4a), suggesting that defeat alone increased microglial density. Knock-down of S1PR3 increased density of IBA1-IR cells in both ND and defeated groups but the highest densities of IBA1-IR cells were exhibited by defeated rats with knock-down of S1PR3 (Fig. 3d and Supplementary Fig. 4a). These findings show that S1PR3 knock-down in socially defeated rats contributes to pro-inflammatory processes in the mPFC and suggest that the elevated S1PR3 in resilient rats buffers inflammatory processes induced by repeated defeat.

To further investigate the substrates of these potential pro-inflammatory processes produced by S1PR3 knock-down, we assessed the expression of two pro-inflammatory cytokines, TNFα and interleukin-1β (IL1β). Compared to ND iAAV-scramble controls, TNFα in both sub-regions of the mPFC was increased in defeated iAAV-scramble rats and this effect was exacerbated in defeated iAAV-S1PR3 rats (Fig. 3e, f and Supplementary Fig. 4b). In the IL, IL1β expression was increased by S1PR3 knock-down regardless of whether rats were defeated or not (Fig. 3h and Supplementary Fig. 4c). These findings with S1PR3 knock-down are corroborated in rats with S1PR3 over-expression. IBA1-IR cell density and TNFα-IR were reduced in the PL and IL of defeated rats over-expressing S1PR3 (AAV1-S1PR3-GFP) compared to defeated rats that were administered control virus (AAV1-GFP) (Fig. 3i–l and Supplementary Fig. 4n, o). Overall, these findings provide evidence that IL1β expression in the mPFC is attenuated by S1PR3 but not influenced by stress but that the expression of TNFα in the mPFC is increased by social defeat and attenuated by S1PR3.

### S1PR3 knock-down promotes vulnerability by increasing TNFα

Based on these results, we hypothesized that the increased anxiety- and depression-like behavior displayed by defeated rats with knock-down of S1PR3 was due to increased expression of TNFα in the mPFC and that knocking down TNFα, but not IL1β, would rescue the anxiogenic and pro-depressive phenotype of rats with reduced S1PR3 in the mPFC. We tested this hypothesis by assessing anxiety-like and depression-like behavior in defeated rats following no knock-down (iAAV-scramble), knock-down of S1PR3 alone (cocktail of iAAV-S1PR3 and iAAV-scramble), knock-down of S1PR3 and TNFα (cocktail of iAAV-S1PR3 and iAAV- TNFα), or knock-down of S1PR3 and IL1β (iAAV-S1PR3 and iAAV-IL1β). A cocktail of two separate viruses was used to ensure robust expression of both siRNA transcripts as packaging multiple transcripts in a single vector can be technically challenging and can result in differential promoter silencing, transcription interference, and unequal levels of gene expression^41^. Knock-down of the targeted genes in the mPFC was effective and no obvious morphological changes were observed (Supplementary Fig. 4d–i). All rats were exposed to repeated social defeat followed by behavioral testing as in Fig. 2a. We reaffirmed that knock-down of S1PR3 in the mPFC (in iAAV-S1PR3-injected rats) increased anxiety-like behavior as assessed by decreased time interacting with the stimulus rat in the social interaction test compared to iAAV-scramble controls (Fig. 3m). When TNFα was also knocked-down at the same time as S1PR3, the increase in anxiety-like behavior was reversed but IL1β knock-down with knock-down of S1PR3 did not produce behavioral changes different from S1PR3 knock-down alone. In the forced swim test, we also confirmed that iAAV-S1PR3 rats displayed increased immobility compared to iAAV-scramble controls. Immobility in iAAV-S1PR3/TNFα rats, but not iAAV-S1PR3/IL1β rats, was significantly reduced compared to iAAV-S1PR3 rats and not statistically different from iAAV-scramble rats (Fig. 3n). Swimming and climbing behaviors were not significantly impacted (Supplementary Fig. 4j). We reaffirmed that S1PR3 knock-down groups displayed decreased restraint-induced ACTH compared to controls, although additional knock-down of TNFα or IL1β had no effects (Fig. 3o). Therefore, behavioral changes caused by TNFα knock-down occurred in the absence of neuroendocrine changes. Together, these results show that the increased anxiety-like and depression-like behavior displayed by rats with S1PR3 knock-down in the mPFC was due to elevated TNFα expression.

We elaborated on these findings by investigating whether pharmacologically inhibiting TNFα signaling could ameliorate the anxiety- and depression-like behavior caused by S1PR3 knock-down. We defeated rats with or without S1PR3 knock-down (S1PR3 expression characterized in Supplementary Fig. 4k, l) and treated them with daily mPFC injections of either saline or the TNFα inhibitor infliximab (50 ng per hemisphere, bilateral) via intracerebral cannulae immediately following social defeat. All rats were exposed to repeated social defeat followed by behavioral testing as in Fig. 2a. We reaffirmed that knock-down of S1PR3 in the mPFC (in iAAV-S1PR3/saline rats) reduced defeat latency, decreased interaction time in the social interaction test, and increased immobility in the Porsolt FST compared to iAAV-scramble/saline controls. These behavioral deficits were rescued by TNFα inhibition as infliximab-treated iAAV-S1PR3 rats were not significantly different from iAAV-scramble rats (Fig. 3p–r). In the Porsolt FST, swimming and climbing behaviors were not significantly impacted, although there was a modest group effect for infliximab-treated rats displaying increased swimming (Supplementary Fig. 4m). These infliximab-mediated behavioral changes occurred in the absence of restraint-induced neuroendocrine changes (Fig. 3s). In sum, both the virally-mediated knock-down of TNFα and its pharmacological inhibition produced similar results. These results indicated that the increased anxiety-like and depression-like behaviors caused by S1PR3 knock-down in the mPFC were due to increased TNFα signaling, which suggests that S1PR3 promotion of behavioral resilience involves inhibition of TNFα signaling in the mPFC.

### S1PR3 elevations in RES rats are induced by stress and mediated by GRs

**Fig. 4:**
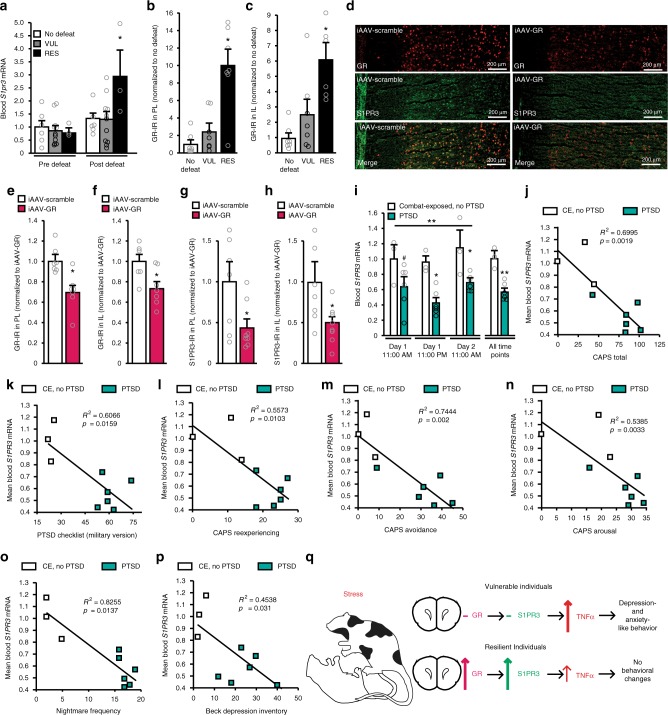
S1PR3 expression is regulated by stress and predicts symptoms of PTSD. **a** Fold *S1pr3* mRNA expression (normalized to pre-defeat ND controls) isolated from tail blood before and after defeat in ND (*n* = 6), VUL (*n* = 11), and RES (*n* = 3) rats. GR-IR (percent area above threshold) in the **b** PL and **c** IL of ND, VUL, and RES rats (*n* = 7/group).  **d** Image of GR and S1PR3 in the mPFC of iAAV-scramble and iAAV-GR rats. Quantification of GR-IR (percent area above threshold) in **e** PL and **f** IL of iAAV-scramble and iAAV-GR rats (*n* = 7/group). S1PR3-IR (percent area above threshold) in **g** PL and **h** IL of iAAV-scramble and iAAV-GR rats (*n* = 7/group). **i** *S1PR3* mRNA expression isolated from whole blood samples was reduced in combat-exposed (CE) veterans with PTSD (*n* = 6 ) compared to CE veterans without PTSD (*n* = 3) at three time points; mean of the three time points was also reduced. Time points are normalized to CE without PTSD mean at 11:00 AM on Day 1. Correlation between mean blood *S1PR3* mRNA expression and **j** Clinician Administered PTSD Scale score, **k** PTSD checklist (military version), **l** re-experiencing symptom cluster of the CAPS score, **m** avoidance symptom cluster of the CAPS score, **n** arousal symptom cluster of the CAPS score, **o** nightmare frequency, and **p** Beck Depression Inventory in combat-exposed veterans with and without PTSD. **q** Model illustrating summary of results: vulnerable rats do not induce GR or S1PR3 expression, allowing social defeat to increase TNFα expression in the mPFC, which contributes to anxiety-like and depression-like behavior. Resilient rats induce GR and S1PR3 expression in the mPFC, thereby attenuating TNFα expression and preventing anxiety-like and depression-like behavior. Bars represent mean + SEM. *p < 0.05, **p < 0.01, #*p* < 0.07. For **a** * indicates Bonferonni post-hoc difference from pre-defeat groups (two-way repeated measures ANOVA). For **b**, **c** * indicates Tukey post-hoc difference from ND control and VUL rats (one-way ANOVA). **e**–**h** Student’s *t*-test. For **i** * and # represent Bonferonni post-hoc differences compared to time-matched controls (two-way repeated measures ANOVA), horizontal bar represents group difference across all three time points

One important question is whether the elevated S1PR3 expression in the mPFC of RES rats occurred as a result of stress experience or is pre-existing. The results above show that defeat induced recruitment of microglia/monocytes and increased TNFα compared to non-stressed rats, suggesting that repeated defeat is necessary to induce these neural changes regulated by S1PR3. Assessment of S1PR3 in the mPFC prior to and following repeated defeat in animals classified as VUL or RES is required to directly answer this question; however there is no current approach that can fully do so. Therefore, we examined S1PR3 expression in blood as a proxy for S1PR3 expression in the brain. We analyzed *S1pr3* mRNA from whole blood samples taken before and after seven days of daily social defeat in ND control, VUL, and RES rats. Prior to defeat, *S1pr3* mRNA expression was the same amongst rats that went on to be VUL or RES to social defeat and in rats that served as ND controls. However, following defeat, RES rats displayed increased *S1pr3* mRNA whereas post-defeat *S1pr3* mRNA in ND control and VUL rats was not different from pre-defeat levels (Fig. 4a). This result suggests that S1PR3 expression, at least in blood, is increased as a function of stress only in rats that have become resilient.

We next asked how S1PR3 expression in the mPFC is increased by stress experience in RES rats. We previously showed that the glucocorticoid response to repeated defeat in RES rats more rapidly approaches levels of novel cage controls whereas VUL rats show protracted glucocorticoid responses to repeated defeat^6^. Glucocorticoid receptors (GR) in the mPFC exert potent negative feedback effects on the neuroendocrine response to stress^42^. We assessed GR in the mPFC of naïve ND control, VUL and RES rats. GR-IR was significantly increased in the PL and IL of RES rats compared to ND control and VUL rats (Fig. 4b, c and Supplementary Fig. 5), suggesting that the increased GRs in RES rats exert negative feedback effects that reduce the glucocorticoid response to repeated defeat in RES rats, as we have previously seen^6^. Binding sites for the GR have been identified proximal to the *S1pr3* gene in rodents^43^ and because GRs are transcription factors that can regulate gene expression, we hypothesized that the increased *S1pr3* expression displayed by RES rats following stress was regulated by GRs. We used iAAV-GR to knock-down GR in the mPFC, exposed rats to seven days of social defeat, and assessed S1PR3 expression. GR knock-down reduced S1PR3 expression in both PL and IL (Fig. 4d–h), indicating that GRs promote increased S1PR3 expression. Together, these findings support the hypothesis that, as exposure to stress continues, activation of GRs in the mPFC, which are in higher density in RES compared to VUL rats, promotes increased expression of S1PR3 in the mPFC and attenuates increases in inflammatory processes including recruitment of microglia/monocytes and expression of the pro-inflammatory cytokine TNFα (Fig. 4q). This confluence of events promotes resilience to stress.

### Blood *S1PR3* mRNA is reduced in PTSD patients

The finding that RES rats exhibited increased S1PR3 in blood suggests that S1PR3 may be a useful blood-based biomarker of resilience/vulnerability to stress in humans. We investigated S1PR3 expression in the blood of combat-exposed U.S. military veterans, some with and others without PTSD (see Supplementary Table 2 for demographics). Compared to combat-exposed controls without PTSD, combat-exposed PTSD patients exhibited significantly reduced or a trend towards reduced *S1PR3* mRNA isolated from whole blood samples analyzed at three consecutive time points 12 h apart as well as a significant reduction in the mean of these three time points (Fig. 4i). Due primarily to the time required for psychiatric evaluation and blood collection, participant recruitment was challenging, particularly for the combat-exposed non-PTSD subject group and for female subjects (only one) in general. Despite the low sample sizes, we found inverse correlations between blood *S1PR3* mRNA and PTSD symptom severity, highlighting the potential of *S1PR3* mRNA as an accurate biomarker for PTSD. The mean *S1PR3* mRNA from these three time points inversely correlated with the total Clinician-Administered PTSD Scale (CAPS-IV) score (Fig. 4j) and with the PTSD Checklist score (Military version, PCL-M, Fig. 4k), an additional, independent measure of PTSD symptoms. Furthermore, *S1PR3* mRNA was inversely correlated with scores on the re-experiencing, avoidance, and arousal symptom clusters on the CAPS and with nightmare frequency on the Nightmare Frequency Questionnaire (Fig. 4l–o). *S1PR3* mRNA was also inversely correlated with scores on the Beck Depression Inventory (Fig. 4p), suggesting that elevated *S1PR3* expression was related to reduced symptoms of depression in these combat-exposed veterans. These findings are consistent with the overall conclusions from the results presented in rats indicating that S1PR3 expression is elevated in resilient individuals. Together, these results identify *S1PR3* as a potential blood-based biomarker of the longitudinal course of symptom severity in individuals with PTSD.

## Discussion

The findings presented in this study are the first to examine in detail the function of S1PR3 in the brain and the first to identify  its role in stress resilience. Here we provide evidence that S1PR3s in the mPFC are necessary and sufficient to promote resilience to the effects of social defeat through attenuation of defeat-induced increases in TNFα in the mPFC. Additionally, we showed that increased mPFC S1PR3 expression was, in part, regulated by GR which were elevated in resilient rats. Furthermore, expression of S1PR3 in blood was increased in resilient rats following stress suggesting that the increase in S1PR3 in mPFC may occur as a function of stress experience and is not pre-existing. Finally, we showed that blood *S1PR3* mRNA was reduced in PTSD patients compared to combat-exposed veterans without PTSD and that *S1PR3* mRNA inversely correlated with PTSD symptom severity.

One potential application of this work in humans is using S1PR3 as a biomarker for the diagnosis and treatment of PTSD and depression. Screening for *S1PR3* mRNA in the blood may assist in the diagnosis of PTSD or influence combat assignments in military personnel. Additionally, *S1PR3* mRNA may be used as a screening tool for anti-inflammatory treatments of depression. Infliximab, which rescued behavioral deficits in S1PR3 knock-down rats, has been shown to reduce symptoms of depression in subgroups of depressed patients with elevated levels of the inflammatory marker C-reactive protein^40^. Screening for blood S1PR3 mRNA may improve accuracy in identifying patients likely to respond to infliximab treatment. Additionally, specifically targeting S1PR3 pharmacologically may have therapeutic potential for treating stress-related psychiatric disorders and/or inflammatory disorders.

There are some important caveats to the interpretations of the observed findings. In defeated rats, mPFC S1PR3 over-expression reduced immobility in the Porsolt FST and S1PR3 knock-down increased immobility. Behavior in the FST, particularly immobility, is highly related to the effectiveness of anti-depressant drugs^44,45^. However, behaviors in the FST can be influenced by a number of factors that are not necessarily directly related to mood disorders^46^ and the FST does not model important symptoms exhibited by humans with depression, such as anhedonia. Although the behavioral findings in the FST were highly reproducible across the experiments presented here and across time in our previous work^6,47^, an expansion of the behaviors influenced by S1PR3 to include clinically relevant functional domains will be important for advancing our understanding of S1PR3 functions. Additionally, it is important to note that adverse effects of social defeat can be attenuated or even negated by group housing rats^48^. The rats in these studies were singly housed, potentially enhancing the impact of social defeat. Finally, the studies here were conducted exclusively or primarily in male subjects. It will be important in future studies to determine whether S1PR3 functions and the mechanisms that underlie these functions are similar in females.

An important finding of this study was that following social defeat, mPFC S1PR3 over-expression caused facilitated ACTH production in response to a challenge restraint exposure whereas S1PR3 knock-down caused attenuated ACTH production. Facilitated responses to novel heterotypic stressors in previously stressed individuals are adaptive, allowing a stressed individual to remain responsive to potential novel stressors in the face of negative feedback, which may reduce responsiveness^49–53^. Indeed, in our previous work, vulnerable rats displayed attenuated ACTH production in response to the heterotypic stress of restraint^6^. GRs^42^, including those in the mPFC^21^, play an important role in negative feedback of the stress response. Increased GR expression in the mPFC of resilient rats may contribute to their ability to more rapidly reduce stress hormone production in response to multiple exposures to social defeat^6^ and grant them an adaptive ability to appropriately respond to a novel, heterotypic stressor (i.e., restraint).

The mPFC has been implicated as a brain region important for negative feedback regulation of the HPA axis^21^ and involved in a wide range of behaviors including attention^30,54^, decision-making^55^, social behavior^56^, memory^57^, fear^35,58^, anxiety^55^, and depression^59^. This diverse functionality of the mPFC may underlie its importance in resilience. Because our original finding of increased *S1pr3* mRNA was from total mPFC lysates and because targeting PL and IL discretely along the dorsal-ventral axis is technically challenging, we over-expressed and knocked-down S1PR3 in both regions. However, we analyzed protein expression in PL and IL separately whenever possible because PL and IL connect to different brain regions and therefore govern different functions. Notable efferent projections of the PL include nucleus accumbens, paraventricular thalamic nucleus, raphe nuclei, and amygdala (central and basolateral nuclei) whereas the IL projects to the BNST and amygdala (medial, basomedial, central, and cortical nuclei)^31^. Indeed, the different connectivity of the PL and IL confers different functionality. In particular, the PL regulates fear expression whereas the IL regulates extinction memory^60^. Defeat and S1PR3 knock-down-induced increases in inflammation were consistently more pronounced in the IL than the PL. Interestingly, neuronal activity in the BNST and basolateral amygdala, regions to which the IL projects, was regulated by S1PR3 in the mPFC in stressed animals. Thus, activation of these downstream structures likely plays an important role in the behavioral and neuroendocrine outcomes regulated by S1PR3 in the mPFC.

We demonstrated that social defeat increased TNFα in the mPFC and that this increase was exacerbated by S1PR3 knock-down. Virally-mediated knock-down or pharmacological inhibition of TNFα signaling in the mPFC rescued behavioral deficits in defeated S1PR3 knock-down rats. Therefore, the increased anxiety-related and depression-related behaviors displayed by defeated S1PR3 knock-down rats was due, at least in part, to increased TNFα. Social defeat and S1PR3 knock-down increased IBA1-IR cell density in the IL, and IBA1-expressing microglia and monocytes represent important sources of TNFα in the brain^61–64^. Thus, it is reasonable to infer that S1PR3 reduces TNFα expression in the mPFC by attenuating stress-induced microglia/monocyte recruitment. However, TNFα is also expressed in neurons^65^ and was increased by social defeat in the PL, where IBA1-IR cell density was unaltered in defeated scramble controls. Therefore, S1PR3 may also play a role in reducing stress-induced increases in neuron-derived TNFα. While the precise mechanism(s) by which S1PR3 attenuates stress-induced increases in TNFα are unknown, the results indicating that TNFα contributed to depression-related and anxiety-related phenotypes are consistent with previous work in rodents^39^ and sub-populations of humans with elevated inflammation^40^.

Together, the findings presented here are the first detailed examination of the function of a sphingolipid receptor in the brain. These studies identified S1PR3 in the mPFC as a critical regulator of resilience to the adverse effects of stress. As a whole, the findings suggest that S1PR3s reduce stress-induced increases in inflammatory cytokines, specifically TNFα, and regulate mPFC network activity to promote resilience to repeated stress. More broadly, the results presented here highlight potential regulatory roles for neuronal sphingolipids and their S1PRs in mediating phenotypes important in stress-related psychiatric diseases. In particular, the finding that *S1PR3* mRNA correlated negatively with PTSD symptom severity may provide the groundwork for the development of treatment strategies targeting sphingolipid receptors for stress-related psychiatric disorders including PTSD, anxiety, and depressive disorders.

## Methods

### Animals

Adult male Sprague–Dawley rats (225–250 g) were obtained from Charles River Laboratories (Wilmington, MA, USA) and served as intruders. Long-Evans retired breeders (650–850 g) served as residents. Rats were singly housed in polycarbonate cages with standard bedding and with food and water available ad libitum. Animals were acclimated to a 12-h light–dark cycle with lights on at 06:15 and lights off at 18:15 in a temperature-controlled vivarium for at least 5 days prior to administration of any stress protocols. All experiments took place during the inactive phase between 1000 and 1400 h. Rats were euthanized by rapid decapitation and their brains were immediately snap-frozen in 2-methylbutane. Each day following social defeat, the rats were inspected by the experimenter and an animal technician. Any signs of pain (blood, limping, etc.) were assessed by a veterinarian who recommended euthanasia if symptoms were too severe. Experiments were performed in compliance with all relevant ethical regulations for animal testing and research. Experiment protocols followed the NIH Guide for the Care and Use of Laboratory Animals and were approved by the Children’s Hospital of Philadelphia Research Institute’s Animal Care and Use Committee.

### Social defeat

The social defeat paradigm was performed as previously described^6^. Rats were randomly assigned to either a social defeat or control group for 5–7 consecutive days. During each episode of social stress, a rat was placed into the home cage territory of an unfamiliar Long-Evans resident previously screened for high aggression. A typical agonistic encounter resulted in intruder subordination or defeat, signaled by the intruder assuming a supine position for 3 s. After defeat, a wire mesh partition was placed in the cage to prevent physical contact between the resident and intruder but allowing visual, auditory, and olfactory contact for the remainder of the 30 min defeat session. Latency to assume a submissive posture (defeat) was recorded and averaged over the seven daily defeat exposures. Rats that were not attacked were not included in defeat latency analysis for that day. If an intruder resisted defeat for 15 min, the resident and intruder were separated with the wire partition for the remainder of the session. Controls were placed behind a wire partition in a novel cage for 30 min daily. Rats were returned to their home cage after each session. To identify VUL or RES rats, the latency of each rat over the course of every day of the defeat paradigm was entered into an R script used to perform cluster analysis on defeat latency averages (code available at www.github.com/cookpa/socialdefeat). The analysis provides probabilities for resilience, with 1 indicating resilience and 0 indicating vulnerability, with 0.5 being the point of delineation between RES and VUL rats^8^.

### Microarray procedure and analysis

A custom-made PCR array (CAPR-10089E, SABiosciences) containing 192 pre-optimized SYBR Green RT PCR assays for 186 genes of interest, 3 housekeeping genes (Beta-actin, Ribosomal protein large P1 and hypoxanthine phosphoribosyltransferase 1) and 3 synthetic control genes (reverse transcription control, positive PCR control and rat genomic DNA contamination control). Samples were run according to manufacturer’s instructions and analyzed as previously described^66^. Three hundred and twenty nanogram RNA per rat was used to synthesize cDNA using the RT^2^ First Strand Kit (SABiosciences). The comparative Ct method was used to plot mRNA expression differences for genes of interest. Ct values were normalized to the average Ct values of the three housekeeping genes for each rat^67^.

### Immunohistochemistry

Brains were sectioned on a cryostat at 20 µm. The following primary antibodies were used: rabbit anti-S1PR3/Edg3 (bs-7541R, 1:100, BIOSS), guinea pig anti-NeuN (ABN90, 1:1000, EMD Millipore), mouse anti-GAD67 (MAB5406, 1:5000, EMD Millipore), goat anti-GFP (ab5450, Abcam, 1:2000), rabbit anti-IBA1 (019-19741, Wako, 1:250), rabbit anti-TNFα (NBP1-19532, Novus Biologicals, 1:100), rabbit anti-IL1β (sc-7884, 1:100, Santa Cruz), goat anti-IBA1 (ab5076, Abcam, 1:100, fluorescent only), chicken anti-GFAP (ab4674, Abcam, 1:5000), and mouse anti-glucocorticoid receptor (ab9568, Abcam, 1:100). The following secondary antibodies were used: goat anti-guinea pig (Alexa Fluor ® 405, Abcam, ab175678), donkey anti-rabbit (Alexa Fluor ® 488, Abcam, ab150073), donkey anti-mouse (Alexa Fluor ® 594, Abcam, ab150108), donkey anti-goat (Alexa Fluor ® 405, ab175664, Abcam), donkey anti-chicken (Alexa Fluor ® 594, Jackson, 703-585-155), and biotinylated donkey anti-rabbit (Jackson Laboratories, 711-065-152). All secondary antibodies were used at a concentration of 1:200. All immunohistochemical comparisons of protein expression were from assays performed at the same time with the same working solutions. For staining with 3,3′-diaminobenzidine (DAB), further amplification was accomplished using Avidin-Biotin Complex (Vectastain). DAB (Sigma) was used as a chromagen. For IL1B immunohistochemistry, an antigen retrieval step was performed by bathing sections in citrate buffer (C9999, Sigma) for 20 min at 95 °C. Quantification of S1PR3 expression in specific cell types (Fig. 1d–h) was performed as follows. Image J was used to open blue (NeuN), red (Gad67), and green (S1PR3) channels from PL and IL images of ND controls, VUL, and RES rats. Randomly selected NeuN+/GAD67− and NeuN+/Gad67+ neurons were marked in dorsal, mid, and ventral regions of all six layers of cortex. Blue and red channels were turned off and the green channel was turned on to assess optical density of S1PR3-IR cells. S1PR3-IR cell count was used to assess S1PR3 knock-down and IBA1-IR cell density. For all other experiments, percent area above threshold (Image J) was used to quantify protein IR. Two sections between Bregma +3.0 and Bregma +3.4 mm were chosen for analysis. Sections in which the tissue was not wholly intact or damaged were discarded from analysis.

### Enzyme-linked immunosorbent assay

Punches from the mPFC were taken from snap-frozen brains for generating tissue lysates. A standard BCA assay was used to quantify total protein concentrations using a Tecan Infinite M200 (562 nm). Sphingosine-1-phospate levels in the mPFC were quantified using a general sphingosine-1-phosphate ELISA kit (MyBioSource, cat. no. MBS2700637). ELISAs were carried out per manufacturer’s instructions. Twenty-five microgram of total protein was added to each well of the ELISA in duplicates. Absolute concentrations of spingosine-1-phosphate from mPFC samples were extrapolated using concentrations of standards.

### Stereotaxic virus injections

The Penn Vector core designed AAV1.CAG.S1PR3.IRES.EGFP.WPRE.SV40 (1.44 × 10^12^ GC/mL) to over-express S1PR3 and AAV1.CB7.CI.eGFP.WPRE.rBG to overexpress eGFP (1.26 × 10^12^ GC/mL). Both CAG and CB7 are non-specific promoters, but AAV1 primarily infects neurons, so the overexpression of S1PR3 and eGFP was likely limited to neurons. AAV constructs expressing siRNA were purchased from Applied Biological Materials. Titers were approximately 1 × 10^9^ GC/mL. A dual convergent promoter system, in which the sense and antisense strands of the siRNA were expressed by U6 and H1 promoters, was used to generate constructs rather than in a hairpin loop to avoid any possible recombination events that can occur. The following siRNA constructs were used: iAAV-scramble (iAAV01501, serotype 1), iAAV-S1PR3 (iAAV03843801, serotype 1), iAAV-TNFα (iAAV06566209, serotype 9), iAAV-IL1β (iAAV06560209, serotype 9), and iAAV-GR (iAAV05783701, serotype 1). For experiments involving the knock-down of S1PR3 and TNFα or IL1β, a 1:1 mixture of iAAV-S1PR3:iAAV-TNFα/iAAV-IL1β was created prior to injection. To ensure that rats in which S1PR3 alone was knocked down received similar amounts of iAAV-S1PR3 compared to double knock-down rats, iAAV-S1PR3 was equally mixed 1:1 with iAAV-scramble prior to injection. Rats were weighed and anesthetized with a ketamine/acepromazine/xylazine cocktail (1/0.2/0.02, 1 mL/kg). The mPFC (A/P: Bregma +3.2 mm, D/V: 4.4 mm, M/L: 0.5 mm) was bilaterally injected with 1.0 µL of virus over the course of 10 min.

### Cannulae surgery and intracerebral drug administration

Immediately following virus injection, rats were fitted with intracerebral cannulae in the same holes drilled for virus injection (A/P: Bregma +3.2 mm, M/L: 0.5 mm). Three additional holes were drilled and bone screws (Plastics One, 0-80) were fastened in the skull (Bregma – 3.5 mm, 5.0 mm left; Bregma – 1.0 mm, 4.0 mm right; Bregma – 8.0 mm, 6.0 mm right) to securely fix the cannulae in place. The bilateral cannulae (Plastic One, C232I/SPC), which allow for a 1 mm projection ventral to their placement, were positioned 3.4 mm ventral to the skull surface. Dental cement was used to adhere the cannulae to the skull and bone screws. Twenty-two gauge double dummy cannulae (Plastics One, C232DC/spc) were placed in the intracerebral cannulae to prevent the cannulae from closing. An infusion dust cap (Plastics One, 303DC/1) was used to cover the cannulae and fasten the dummy cannulae. Immediately following social defeat, the dummy cannulae were removed and a hydraulic syringe filled with saline was used to inject 1 µL of either saline or infliximab (Remicade ®, Janssen Immunology, 100 ng/2 µL total) in awake rats. Dummy cannulae were replaced and the rats were returned to their homecage.

### Social interaction

Animals were placed in an open field black box (70 cm × 70 cm) with an age-matched stimulus rat of the same strain and of a similar size and allowed to interact for 15 min. Time interacting with the stimulus rat was defined by the time the rat was actively investigating the stimulus rat with its snout closer than 3 cm away (approximately the length of the snout of the rat) from the stimulus rat. Each interaction was videotaped and coded for social interaction time by 2 coders who were blind to the experimental conditions.

### Porsolt forced swim test

Rats were placed in a glass cylinder filled with 60 cm of water so that their tails could not touch the bottom of the cylinder while floating. Rats underwent a 15 min training phase followed by a 5 min test phase on the following day. The test phase was videotaped and coded for time engaged in immobile, swimming, and climbing behaviors by 2 coders who were blind to the experimental conditions.

### Restraint stress paradigm

Rats were restrained for 30 min and returned to their homecage for an additional 30 min prior to sacrifice. Tail blood was taken (~400 µL) at 0 min, again at 15 min and 30 min (during restraint), and at 60 min (during euthanization). Plasma adrenocorticotropic hormone (ACTH) was assayed with a radioimmunoassay kit from MP Biomedical (Orangeburg, NY, USA). The minimum level of detection for ACTH was 5.7 pg/mL.

### Local field potential recordings

The left mPFC was injected with iAAV-scramble or iAAV-S1PR3 as described above to achieve S1PR3 knockdown (A/P: Bregma + 3.2 mm, D/V: 4.4 mm, M/L: 0.5 mm left) with a recording electrode placed 0.5 µM ventral to the injection site. Recordings were performed on the first and fifth days of the experimental manipulation. Cables connected the head stage to the data acquisition system and pre-defeat baseline recordings were done in the intruder’s home cage. The cables were then disconnected and the rat was placed in the resident’s cage. Post-defeat recordings occurred after the physical interaction, while the intruder was in resident’s cage but was physically separated from the resident by the wire partition, which maintained visual, olfactory, and auditory communication between resident and intruder. Electrode recordings in the mPFC were amplified at a gain of 5000 Hz, bandwidth of 1–150 Hz. mPFC raw traces were time stamped in Spike2 to remove noise and converted to Power Spectra Density (PSD) plots indicating the relative power in 128 frequency bins from 0 to 50 Hz using Neuroexplorer (Nex Technologies, Madison, AL).

### Blood collection and isolation of mRNA from whole blood

Four hundred microliter of tail blood was collected in RNAprotect Animal Blood Tubes (Qiagen, cat. no. 76554) 1 day before and 1 day after 7 days of social defeat. For rat, blood mRNA was isolated using the RNeasy Protect Animal Blood Kit (Qiagen, cat. no. 73224) according to the manufacturer’s instructions. For human, blood mRNA was isolated using the PAXgene blood RNA Kit (Qiagen, PreAnalytix, cat. no. 762164) according to the manufacturer’s instructions.

### qRT-PCR

Reverse transcription was performed using a High-Capacity cDNA Reverse Transcription kit (4368814, Thermo Fischer Scientific). qPCR was performed with an ABI 7500 PCR machine using SYBR Green as a fluorophore. Primers used to amplify cDNA were rat *Gapdh* (forward: 5′-AGACAGCCGCATCTTCTTGT-3′, reverse: 5′- CTTGCCGTGGGTAGAGTCAT-3′), rat *S1pr3* (forward: 5′-CCTCATCACCACCATCCTCT-3′, reverse: 5′-CCCTGAGGAACCACACTGTT-3′), human *GAPDH* (forward: 5′-GGATTTGGTCGTATTGGG-3′, reverse: 5′-GGAAGATGGTGATGGGATT-3′), human *S1PR3* (forward: 5′- TCATCTGCAGCTTCATCGTC-3′, reverse: 5′- CGTCTTCTTGCCAGACATCA-3′).

### Psychiatric assessments and blood collection

Subjects were veterans of Operation Enduring Freedom, Operation Iraqi Freedom, or Operation New Dawn (OEF/OIF/OND). All had combat exposure during their military deployment. The study was approved by the Institutional Review Board of the Corporal Michael J. Crescenz VA Medical Center. All subjects provided informed consent. Subjects were assessed for current DSM-IV PTSD using the Clinician Administered PTSD Scale (CAPS), which was used to characterize them as with or without current PTSD. Subjects completed a variety of self-report scales including the PTSD Checklist—Military Version (PCL-M), Nightmare Frequency Questionnaire (NFQ), and Insomnia Severity Index (ISI). Subjects were admitted to the Center for Human Phenomic Science for a 48-hour inpatient stay. An indwelling catheter was placed and blood samples (5 mL) were taken every 2 h for 48 h in PAX gene tubes to ensure stabilization of RNA. Of these samples, 3 samples taken 12 h apart (beginning with the first collection at 11:00 AM on Day 1) were analyzed. mRNA was isolated from whole blood samples using the PAXgene Blood RNA Kit (PreAnalytix, Qiagen, cat. no. 762164) and processed per the manufacturer’s instructions.

### Correlations between *S1PR3* mRNA and psychiatric data

Statistical analyses were performed using R Model selection to minimize AIC. Repeated Measures ANOVA was performed for S1PR3 dCT value vs. each PTSD assessment across 0, 12, and 24 h time points. Multiple-testing correction was performed using the positive false discovery rate method. Results of analyses are presented in Supplementary Table 3. Repeated measures ANOVA was performed using the gls function in nlme package (version 3.1-137) in R (version 3.5.0). Time of assessment and time vs assessment interaction were not found to be significantly associated with any assessment after multiple testing correction. Comparisons of gls models were made with available demographic covariates alone or in combination using age, race, marital status, employment, and education. The lowest model AICs across all responses were from four models: those using age alone, race alone, age and race together, or no demographic covariates. A comparison of AIC of these four models showed that the model using age alone had the best overall fit for all assessments, with ΔAIC < 2 of age alone vs best fit, except for BDI13, CAPS8, NDQ11, CAPS10, PCLM_11, and PCLM_10, which were better supported by a model using race alone as the demographic covariate.

The final equation in R that was used was:

model_gls = gls(dCT ~ *x* * Time + Age, correlation = corAR1(form = ~ Time | SubID), data = data, method = “REML”, na.action = na.omit) where dCT is the S1PR3 dCT value, *x* is the assessment variable such as CAPS_avoidance or BDI_20. Age is the reported subject age, Time is 0, 12, or 24 h (as factors) and SubID is the subject ID.

Due to the expected and observed correlations between the related assessments, multiple testing corrections for the repeated measures ANOVA results on the 118 responses were performed using the positive false discovery rate and reported as the Bayesian posterior *p*-value (*q*-value) using the qvalue package in R (version 2.12.0).

### Statistical analyses

Statistical analyses were performed using Prism 5 and 7. Raw means are presented in Source Data. Differences between means were assessed using a two-tailed, unpaired Student’s *t*-test unless otherwise indicated. Differences among three or more means were assessed using one-way ANOVA and Tukey post-hoc tests. Two-way ANOVA using Bonferroni or Student-Newman-Keuls post hoc tests were used to assess differences in analyses with two variables. Repeated-measures two-way ANOVA with Bonferroni post hoc tests were used for analyzing data analyzed over multiple time points (e.g., 5 min binds in social interaction, pre-defeat vs. post-defeat blood samples). Regression analysis was used to detect correlations. Detailed results of all statistical analyses are listed in Source Data. Data beyond three standard deviations from the mean were considered outliers and discarded from analysis. All data presented in a single panel were collected and analyzed as a single cohort with the exception of the double knock-down experiments presented in Fig. 3g–i and Supplementary Fig. 3d–g, which were collected in two separate cohorts.

### Reporting summary

Further information on research design is available in the Nature Research Reporting Summary linked to this article.

## Supplementary information

Supplementary Information

Reporting Summary

Source Data

## Data Availability

Individual data points are graphed in each main and supplementary figure. Source data files that support the findings of this study are available from the corresponding author upon request and at: https://figshare.com/articles/Corbett_et_al_2019_S1PR3_promotes_stress_resilience_source_data/8194148. Data summaries from the custom PCR array are available in Supplementary Table 1. Figshare 10.6084/m9.figshare.8194148
