# Supplementary material for: Sphingosine-1-phosphate receptor 3 in the medial prefrontal cortex promotes stress resilience by reducing inflammatory processes
Source: Nat Commun. 2019 Jul 17;10:3146. doi: 10.1038/s41467-019-10904-8 (PMC6637233; doi:10.1038/s41467-019-10904-8)
Supplement: Supplementary file 1 — Supplementary Information [file 41467_2019_10904_MOESM1_ESM.pdf]

Supplementary Information

Sphingosine-1-phosphate receptor 3 in the medial prefrontal cortex promotes stress resilience by reducing inflammatory processes

Corbett et al.,

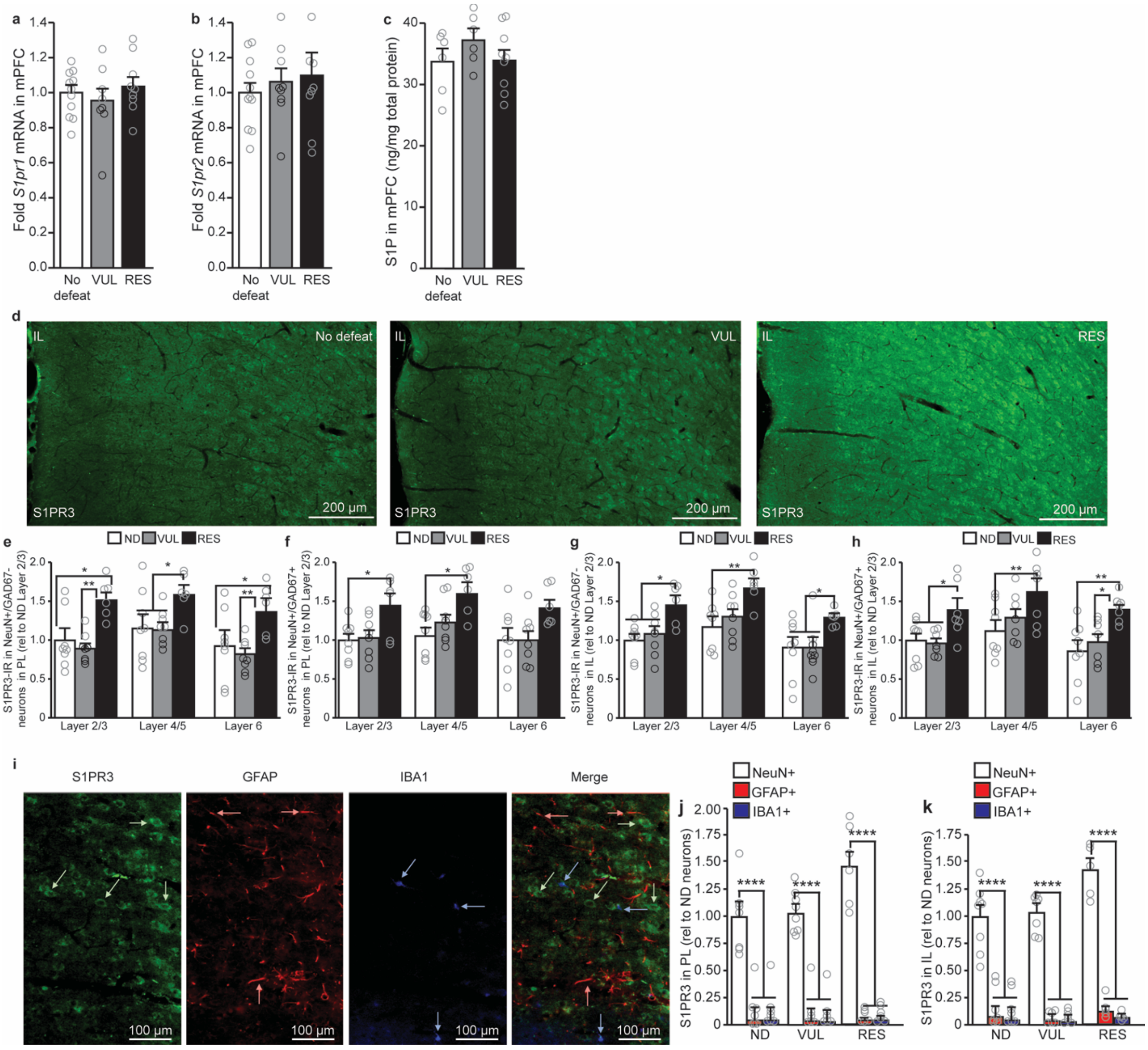

Supplementary Figure 1: *S1pr1* and *S1pr2* mRNA is not altered in the mPFC of resilient rats. (**a**) Fold mPFC *S1pr1* mRNA (relative to ND controls) in ND (n=11), VUL (n=9), and RES (n=9) rats. (**b**) Fold mPFC *S1pr2* mRNA (relative to ND controls) in ND (n=11), VUL (n=9), and RES (n=9) rats. (**c**) Sphingosine-1-phosphate levels (ng per mg of total protein) in the mPFC of ND (n=6), VUL (n=6), and RES (n=9) rats. (**d**) Image of S1PR3 in the IL of ND, VUL, and RES rats. Layer specific comparison of S1PR3 expression in (**e**) excitatory neurons in PL, (**f**) inhibitory neurons in PL (**g**) excitatory neurons in IL, and (**h**) inhibitory neurons in IL of ND (n=8), VUL (n=7), and RES (n=6) rats. (**i**) Image of S1PR3 expression in GFAP+ and IBA1+ glia. Quantification of S1PR3 expression in neurons (NeuN+), astrocytes (GFAP+), and microglia/monocytes (IBA1+) in the (**j**) PL and (**k**) IL of ND (n=8), VUL (n=7), and RES (n=6) rats. Tukey's post-hoc comparisons following one-way ANOVA. *$p < 0.05$, **$p < 0.01$, ****$p < 0.0001$. Bars represent mean + SEM.

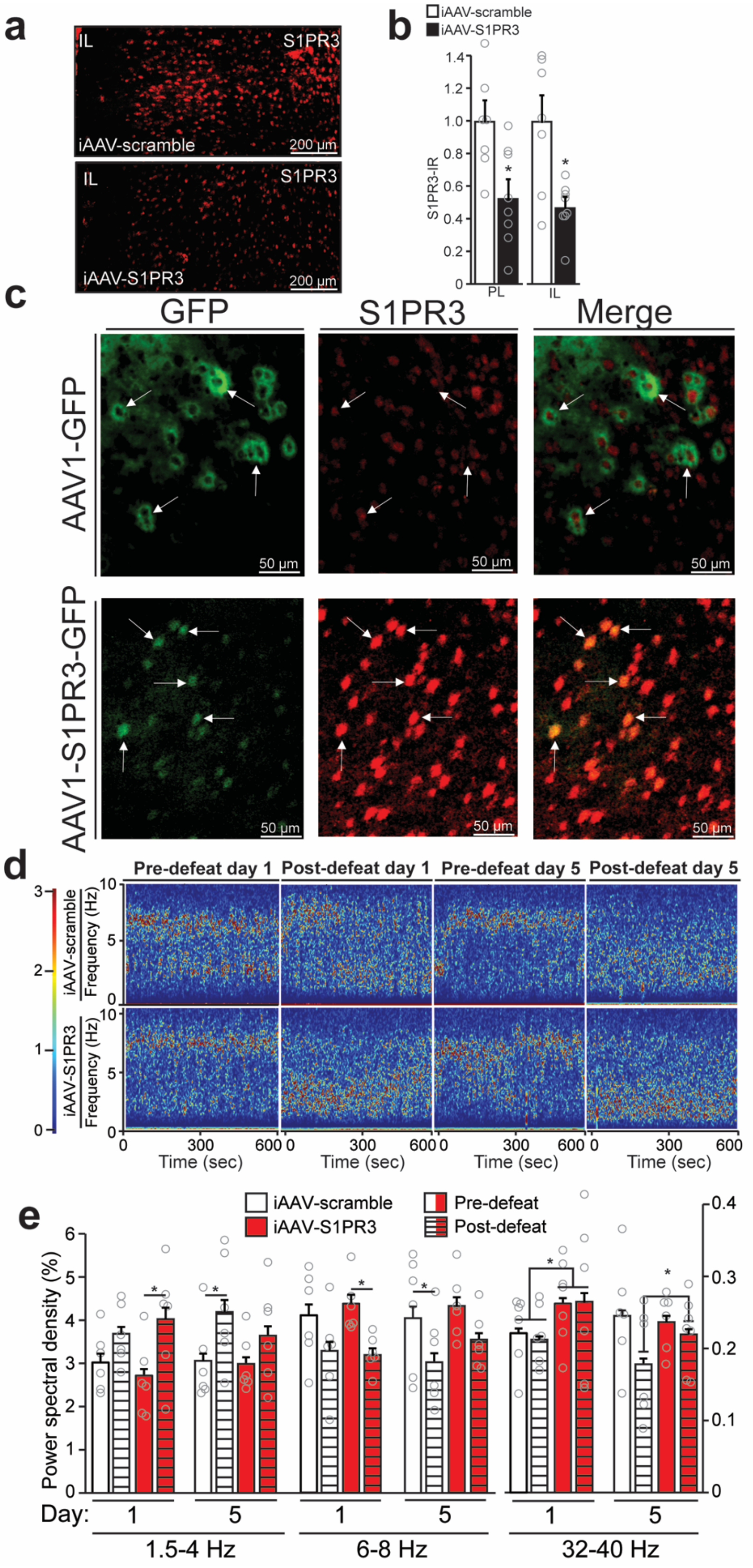
a
IL
S1PR3
iAAV-scramble
200 µm
IL
S1PR3
iAAV-S1PR3
200 µm
b
iAAV-scramble
iAAV-S1PR3
S1PR3-IR
PL
IL
c
GFP
S1PR3
Merge
AAV1-GFP
AAV1-S1PR3-GFP
50 µm
d
Pre-defeat day 1
Post-defeat day 1
Pre-defeat day 5
Post-defeat day 5
iAAV-scramble Frequency (Hz)
iAAV-S1PR3 Frequency (Hz)
Time (sec)
e
iAAV-scramble
iAAV-S1PR3
Pre-defeat
Post-defeat
Power spectral density (%)
Day:
1.5-4 Hz
6-8 Hz
32-40 Hz

Supplementary Figure 2: S1PR3 knockdown causes changes in mPFC network activity. (**a**) Image and (**b**) quantification of S1PR3 expression in the PL and IL 7 days following injection with either iAAV-scramble (n=7) or iAAV-S1PR3 (n=8), analyzed using Student's t-test. (**c**) Representative images of GFP (green) and S1PR3 (red) in the IL of AAV1-GFP and AAV1-S1PR3-GFP rats. Image demonstrates increased S1PR3 expression following overexpression. (**d**) Representative spectrogram for power spectral density percentages in iAAV-scramble and iAAV-S1PR3 rats over a 10 min recording session at pre-defeat day 1, post-defeat day 1, pre-defeat day 5 and post-defeat day 5 time points. (**e**) Pre- and post-defeat power spectral density percentages on days 1 and 5 of the social defeat paradigm in iAAV-scramble and iAAV-S1PR3 rats (n=6/group). Bars represent means + SEM. *$p < 0.05$. Horizontal bars represent Bonferonni post-hoc differences following two-way repeated measures ANOVA.

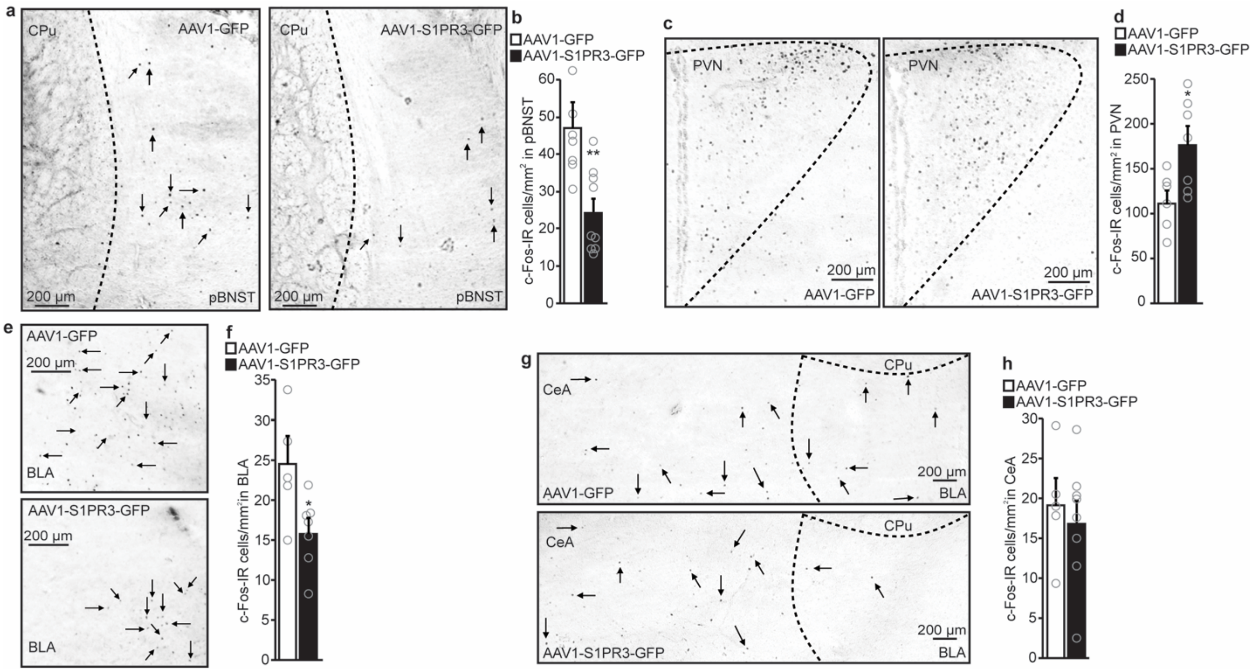

Supplementary Figure 3: S1PR3 overexpression in the mPFC alters neuronal activity markers in targeted brain regions

AAV1-GFP and AAV1-S1PR3-GFP rats that underwent social defeat and behavioral testing were sacrificed 60 min following the onset of a 30 min restraint. (**a**) Image and (**b**) quantification of c-Fos in the pBNST. (**c**) Image and (**d**) quantification of c-Fos in the PVN. (**e**) Image and (**f**) quantification of c-Fos in the BLA. (**g**) Image and (**h**) quantification of c-Fos in the CeA. AAV1-GFP n=7, AAV1-S1PR3/GFP n =9. Bars represent mean + SEM. *$p < 0.05$, **$p < 0.01$, analyzed using Student's t-test. Arrows point to c-Fos-IR cells. pBNST = posterior subdivision of the bed nucleus of the stria terminalis, PVN = paraventricular nucleus of the hypothalamus, BLA = basolateral amygdala, CeA = central amygdala, CPu = caudate putamen.

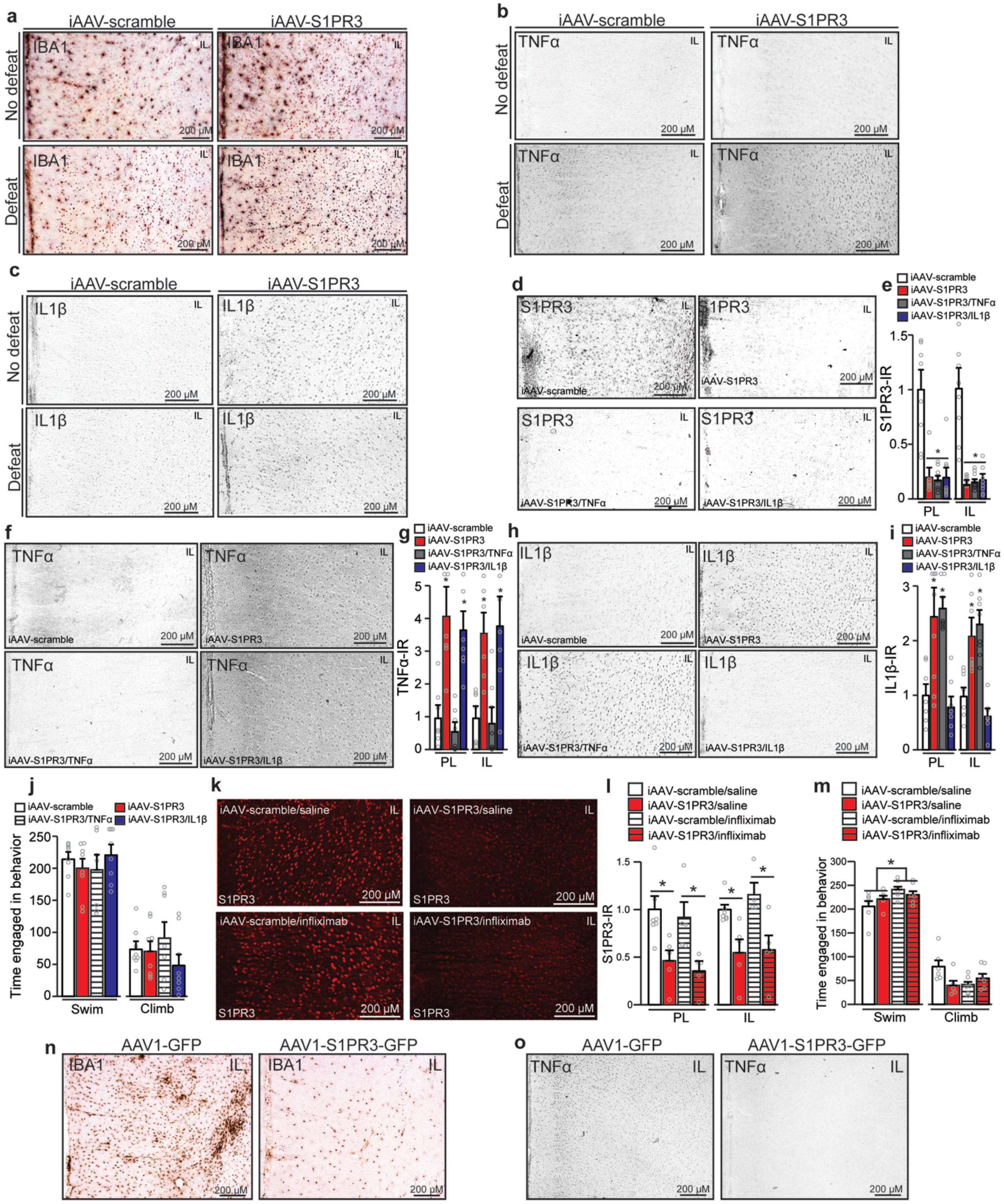
a
iAAV-scramble
iAAV-S1PR3
No defeat
Defeat
IBA1
IL
200 μM
b
TNFα
c
IL1β
d
S1PR3
iAAV-scramble
iAAV-S1PR3
iAAV-S1PR3/TNFα
iAAV-S1PR3/IL1β
e
S1PR3-IR
PL
IL
f
g
TNFα-IR
h
i
IL1β-IR
j
Time engaged in behavior
Swim
Climb
k
iAAV-scramble/saline
iAAV-S1PR3/saline
iAAV-scramble/infliximab
iAAV-S1PR3/infliximab
l
m
n
AAV1-GFP
AAV1-S1PR3-GFP
o

Supplementary Figure 4: S1PR3 attenuates stress-induced inflammation in the mPFC. (**a**) Images of IBA1-IR cells in the IL demonstrating that IBA1-IR cell density is increased by social defeat, S1PR3 knock-down, and the combination of social defeat and S1PR3 knock-down. (**b**) Images of TNFα-IR in the IL demonstrating that TNFα-IR is increased by social defeat, an effect that is exacerbated by S1PR3 knock-down. (**c**) Images of IL1β-IR in the IL demonstrating that IL1β is increased by S1PR3 knock-down, but not social defeat. Images (**d**) and quantification (**e**) confirming S1PR3 knock-down in the PL and IL in all knock-down groups (iAAV-S1PR3 n=7, others n=8/group). Images (**f**) and quantification (**g**) demonstrating that TNFα expression is reduced in the PL and IL of iAAV-S1PR3/TNFα rats compared to iAAV-S1PR3 and iAAV-S1PR3/IL1β rats (iAAV-S1PR3 n=6, others n=7/group). Images (**h**) and quantification (**i**) demonstrating that IL1β expression is reduced in the PL and IL of iAAV-S1PR3/IL1β rats compared to iAAV-S1PR3 and iAAV-S1PR3/TNFα rats (iAAV-S1PR3 n=7, others n=8/group). (**j**) Time swimming and climbing in the Porsolt FST is unchanged among knock-down groups (iAAV-S1PR3/TNFα n=7, others n=8/group). Image (**k**) and quantification (**l**) of S1PR3 in the PL and IL indicating that compared to iAAV-scramble control rats, S1PR3-IR is reduced in iAAV-S1PR3 rats regardless of treatment with either saline or infliximab (iAAV-scramble/saline n=7, others n=5/group). (**m**) Quantification of time engaged in swim and climb behaviors in the Porsolt forced swim test (iAAV-S1PR3/saline n=5, others n=6/group). (**n**) Image demonstrating reduced IBA1-IR cell density in the IL of AAV1-S1PR3-GFP rats compared to AAV1-GFP rats. (**o**) Image demonstrating reduced TNFα-IR in the IL of AAV1-S1PR3-GFP rats compared to AAV1-GFP rats. Bars represent mean + SEM. *$p < 0.05$. For (e,g, and i), * represents statistical difference from control group as assessed by Tukey's post-hoc test following one-way ANOVA. For (l,m), * represents statistical difference as assessed by Bonferonni post-hoc test following two-way ANOVA.

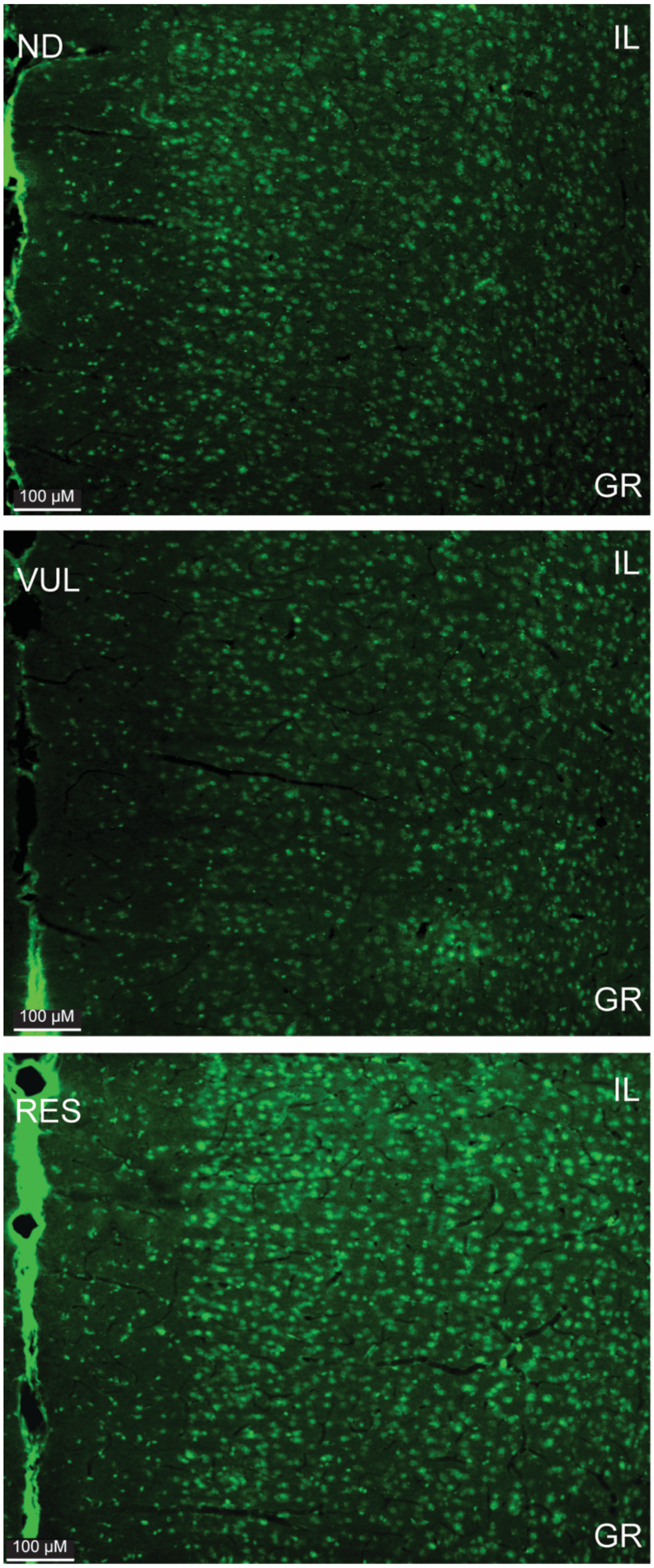

Supplementary Figure 5: GR expression is increased in the mPFC of resilient rats. Representative images of GR expression in the IL of non-defeated controls, vulnerable, and resilient rats.

Supplementary Table 1: Targeted PCR array summary (No defeat n = 11, VUL n = 9, RES n = 9)

							p-values		
Gene Symbol	ND mean	ND SD	VUL mean	VUL SD	RES mean	RES SD	ND vs. VUL	ND vs. RES	VUL vs. RES
Cyp11b1	1	0.1922192	0.866382	0.194374	1.01423	0.26383	0.1412267	0.88838046	0.186462386
Dnmt3a	1	0.2329242	1.0270233	0.205633	1.0459228	0.17212	0.7888757	0.61640848	0.829979951
Dnmt3b	1	0.5687209	1.0863876	0.485235	1.3633596	0.43847	0.7227102	0.12023604	0.20847704
Kat5	1	0.1097765	0.8569089	0.139333	1.0207438	0.14874	0.0192025	0.71837688	0.02442219
Il6st	1	0.1386066	0.8807375	0.232635	1.0349116	0.20242	0.171581	0.64718639	0.140724727
Hat1	1	0.0633466	0.8987138	0.119991	0.9286378	0.12057	0.026	0.1013479	0.595268339
Stat2	1	0.1370544	0.974808	0.131048	0.9551511	0.21275	0.6816283	0.56876434	0.813966403
Tfg	1	0.0914943	0.8536089	0.175106	0.9746607	0.14049	0.0269206	0.62664118	0.11316194
Stat4	1	0.6539433	0.5583707	0.191182	0.7737667	0.30514	0.0668929	0.33083003	0.08673285
FosB	1	0.2548338	0.9908179	0.215455	1.0613917	0.28932	0.9325832	0.61104841	0.558272666
Ube2a	1	0.1111601	0.9364579	0.176519	0.9510348	0.13134	0.3389763	0.36650453	0.839492492
Prmt7	1	0.0824353	1.0059156	0.080026	1.0534425	0.16996	0.8733124	0.36351448	0.455036842
Prmt2	1	0.252596	1.0194448	0.240027	0.9064531	0.10648	0.862966	0.2915825	0.194279934
Hdac1	1	0.1588702	0.8655674	0.177094	0.8487879	0.11735	0.0905104	0.02369705	0.808674836
Narg1	1	0.1140349	0.9487214	0.142794	1.007957	0.16857	0.3831361	0.899647	0.422825105
Narg2	1	0.1192583	0.8961598	0.121984	0.940226	0.16476	0.0711679	0.34954693	0.520607735
Adrb2	1	0.228497	0.9287771	0.254607	1.0880733	0.28084	0.5182292	0.43836132	0.214417528
Agtr2	1	0.8333444	1.8498775	1.261482	1.30832	0.57663	0.0760937	0.36012517	0.235283036
Bdnf	1	0.2187928	0.9239307	0.282677	1.0299886	0.34135	0.505711	0.81131894	0.473818281
Drd1a	1	0.2414626	1.1412544	0.328728	0.8778128	0.19677	0.2823565	0.22187925	0.046558747
Egr1	1	0.2586438	0.7910699	0.285125	0.8107508	0.29419	0.1031422	0.13314501	0.884297717
Nr3c1	1	0.1373024	0.9739679	0.140657	0.934392	0.12355	0.6814194	0.26582172	0.522404493
Il6	1	0.2297455	834707381	2.5E+09	198707745	6.3E+08	0.3050558	0.33056493	0.446867944
Ngfr	1	0.2827961	1.296908	0.363577	1.1084284	0.67826	0.0473928	0.63205458	0.463040633
Nos2	1	0.8732821	0.8963361	0.745643	1.2590575	0.45926	0.7814589	0.41277082	0.213620006
Oprd1	1	0.293707	1.107821	0.34743	0.9974774	0.26458	0.4613647	0.98424266	0.458159083
Serpine1	1	0.5187075	1.1663964	0.793568	0.7864432	0.44889	0.5790614	0.32805963	0.210087113
Tacr1	1	0.3097853	1.1287636	0.440981	1.04219	0.17768	0.4533991	0.7100617	0.574313584
Tgfa	1	0.368552	0.8737615	0.280225	0.9508022	0.4009	0.408961	0.77265164	0.63736742
Tnf	1	0.5046631	0.8827954	0.358691	1.0443917	0.33036	0.5657905	0.816157	0.320903438
Ucp1	1	0.7579789	0.9708578	0.606333	1.075122	0.57892	0.9266693	0.80284474	0.706226271
Cckar	1	0.5847253	1.5348055	1.071167	0.9575908	0.86286	0.15405	0.89559534	0.195719018
Adrb1	1	0.1918371	0.9300636	0.197037	1.0304644	0.21678	0.4333681	0.73630475	0.307540038
Grpr	1	0.2080627	1.0804379	0.264586	0.9008381	0.32664	0.455948	0.41238323	0.208584442
Calcrl	1	0.175156	1.0747534	0.175269	1.087536	0.18645	0.3550616	0.28112041	0.8798334
Ntrk2	1	0.1503629	0.9291751	0.129551	1.020926	0.18743	0.2800702	0.77986063	0.236624826
Stat3	1	0.1383679	1.0976727	0.128211	1.104628	0.16452	0.1221184	0.13004317	0.920022071
Gfra2	1	0.1391591	0.9151868	0.226704	1.0316312	0.17788	0.3169164	0.65340743	0.22731864
Il10	1	0.8812283	0.6500847	0.338504	0.8093877	0.4395	0.417566	0.7504914	0.392549759
Oxtr	1	0.540386	1.3765679	0.8221	1.0159492	0.4231	0.2338755	0.94119802	0.238546566
Tshr	1	0.3003275	0.7479545	0.554844	0.8406386	0.35966	0.2110112	0.28252281	0.667812284
Vcam1	1	0.1516672	1.0090137	0.252794	1.1130264	0.24378	0.9223719	0.21277986	0.374226174

Arrb1	1	0.1357808	1.027901	0.218364	1.0293982	0.15024	0.730312	0.64291173	0.986185009
Arrb2	1	0.1044171	1.0385391	0.267077	1.0371767	0.16312	0.664282	0.53731408	0.989320871
Fgf9	1	0.1216224	0.9505431	0.252258	1.0649252	0.12584	0.57178	0.24419375	0.220651234
Galr1	1	0.6655895	1.5983819	0.630258	1.2104414	0.7876	0.0554584	0.51500737	0.255740388
Icam1	1	0.2505199	1.0814925	0.221752	0.9781471	0.33639	0.4563479	0.86686654	0.445831414
Lhcgr	1	0.6655792	0.4120582	0.517346	0.4733201	0.56564	0.0440714	0.0669293	0.809127009
Oxt	1	0.7030098	1.0790189	0.857012	1.3291373	1.00753	0.8231536	0.39244021	0.569989786
Ywhaz	1	0.1813707	0.9484746	0.225261	1.0364514	0.17399	0.5774869	0.64446521	0.351238884
Pspn	1	0.2425634	0.9405484	0.356656	1.0904079	0.35396	0.6631695	0.49944513	0.371377931
Hcrtr1	1	0.2754007	1.095089	0.30962	1.154522	0.39513	0.4767283	0.30778594	0.721904492
Hcrtr2	1	0.2274144	0.8830328	0.28219	0.8778419	0.19492	0.3177133	0.20422619	0.962999655
Il1r1	1	0.1772791	1.0664725	0.43055	1.1978799	0.49358	0.6453794	0.22762261	0.546654422
HcRt	1	0.4429377	0.9964848	0.509403	0.8283482	0.26685	0.987007	0.3018026	0.372795042
Ntf4	1	0.6184707	0.8433905	0.486328	0.9049264	0.53901	0.5793905	0.70898615	0.808928661
Kcnj6	1	0.2409758	0.8882081	0.229715	1.0853241	0.19601	0.3059432	0.38756298	0.059598061
Avp	1	1.0760466	0.4834052	0.621216	0.9091983	1.19904	0.2242325	0.86194208	0.331565305
Bcl2	1	0.1075586	0.9854806	0.136649	1.008171	0.14676	0.7931125	0.88505846	0.732446167
Casr	1	0.6304671	0.8876667	0.511757	0.6980426	0.35514	0.6720288	0.19842629	0.357022579
Grin1	1	0.2222111	1.0278646	0.169714	1.0378556	0.11933	0.7608144	0.63749356	0.882746413
Grm1	1	0.1479232	1.0229336	0.14985	1.1057367	0.22913	0.7356133	0.21987631	0.370379196
Il6ra	1	0.1265074	1.0049328	0.347925	1.0571854	0.15129	0.9655213	0.35753936	0.670684901
Bax	1	0.1259386	1.0160515	0.195274	1.0090986	0.09462	0.8264207	0.85472782	0.921145199
Cyp19a1	1	0.3207272	0.9578846	0.357972	0.9014351	0.36121	0.7846371	0.51560742	0.736862829
Oprk1	1	0.1695426	1.158308	0.460153	1.1054457	0.14141	0.3024567	0.14037292	0.73319526
S1pr2	1	0.1955874	1.0569538	0.24004	1.0965073	0.39424	0.5655763	0.47947383	0.797780858
Avpr1b	1	0.7970534	0.603734	0.470703	0.5775345	0.32913	0.2059385	0.13599128	0.888874125
Ptgs2	1	0.1833984	1.0486011	0.19386	1.0473213	0.25488	0.5725432	0.6284154	0.990402886
Gria2	1	0.1133978	1.0129666	0.1767	1.060294	0.12659	0.844511	0.26376776	0.507763654
S1pr1	1	0.1331851	0.9530362	0.198899	1.0413072	0.15086	0.5360941	0.51307516	0.287910298
Gnas	1	0.0569279	0.8899692	0.165938	1.0167044	0.08375	0.0535163	0.59604939	0.047495502
Gdnf	1	0.3670985	0.885589	0.348151	1.2329089	0.60497	0.4871395	0.29431567	0.149554016
Ucn	1	0.8096975	0.8376598	0.416875	0.701977	0.31274	0.8136611	0.45793054	0.43023359
Galr2	1	0.399587	1.0347687	0.169905	1.0351179	0.46966	0.8109339	0.85510375	0.998344955
Fgf2	1	0.3956795	0.9241933	0.205412	0.9654256	0.36076	0.610296	0.83706455	0.766900495
Pthr1	1	0.1594077	1.0633691	0.344803	1.2309339	0.53658	0.5925385	0.18795687	0.435354843
Tgfb1	1	0.1760377	1.011623	0.153568	1.0878943	0.21374	0.8782496	0.31474888	0.389172972
Ntrk1	1	0.6227871	1.7925496	1.293194	1.1510188	1.03951	0.1057562	0.70674499	0.292584577
Jun	1	0.1489823	0.9318446	0.174034	1.0348432	0.19444	0.3575762	0.64821374	0.24248089
Junb	1	0.2659176	1.2286837	0.26718	1.1682757	0.28876	0.0722954	0.1804475	0.643241486
Lif	1	0.4863666	1.56192	1.238024	0.9224563	0.40833	0.1861008	0.70798502	0.162892606
Fos	1	0.244795	1.0830761	0.285183	0.9561084	0.26742	0.4920086	0.69886556	0.330628244
Cxcr4	1	0.2378596	1.2935741	0.45738	1.2354363	0.25468	0.0805283	0.04114792	0.732672925
Max	1	0.12132	1.019499	0.126611	1.009813	0.14048	0.7298778	0.86543098	0.876965157
Ctgf	1	0.3097524	0.9355712	0.440966	0.9636566	0.43664	0.7058549	0.82693952	0.890818668
Adcy5	1	0.1328103	1.1301411	0.21515	1.1128932	0.11786	0.1139474	0.05427149	0.82855692
Grm4	1	0.2440898	1.0990104	0.207621	1.1719345	0.23367	0.3480044	0.11641115	0.483955718
Crhr2	1	0.3177672	1.1806546	0.255373	1.1977254	0.52118	0.1851109	0.30185747	0.930166745

Fgfr1	1	0.1739554	1.0798335	0.184783	1.1088534	0.13558	0.3338146	0.12894173	0.699045054
Prmt1	1	0.10498	0.9861744	0.164212	1.0636465	0.11657	0.8217632	0.20356602	0.248204517
Tp53	1	0.1749224	1.1394544	0.253414	1.0315328	0.15034	0.1631873	0.66436371	0.268646932
Vgf	1	0.2611961	1.148959	0.350307	1.0336125	0.23751	0.2900818	0.7618834	0.408146053
Crhr1	1	0.1528404	1.0423452	0.244942	0.9830052	0.30223	0.6417748	0.87067779	0.646665692
Agtr1b	1	0.7641379	0.8958913	0.63521	1.1205029	1.37416	0.7278756	0.8378732	0.677417001
Atf2	1	0.0919913	0.9476879	0.196135	1.0315366	0.13391	0.440781	0.53343563	0.287280726
Crh	1	0.1791051	0.815889	0.248441	1.0514273	0.19254	0.0701077	0.53351737	0.032801785
Gmfb	1	0.3161963	0.9155605	0.214367	1.0358292	0.16694	0.958926	0.27229437	0.187785546
Gnaq	1	0.1458044	0.9245115	0.190483	1.0672495	0.15974	0.3282931	0.32572954	0.093568999
Mmp9	1	0.1991648	1.1263927	0.449698	1.2520134	0.55291	0.4116121	0.17267744	0.596675944
Ntf3	1	0.3266075	0.9474346	0.258565	1.09398	0.25527	0.6996087	0.47478088	0.231141625
Pdpk1	1	0.1465928	0.9021525	0.166051	1.0993731	0.15991	0.1786298	0.15366513	0.017331908
Ptger2	1	0.2751016	1.0805706	0.340422	0.9843648	0.26012	0.5650996	0.89522666	0.495485119
Sctr	1	0.3969032	0.8276549	0.625946	0.7713148	0.35674	0.4631734	0.18266388	0.809858548
Ube2b	1	0.0803589	0.988284	0.141247	1.0199908	0.07392	0.8179539	0.56128319	0.541749649
Il1b	1	0.558363	1.1799029	0.676951	1.1586892	0.79192	0.2063641	0.26537946	0.950981446
Ccl2	1	0.7228888	1.1622953	1.162161	0.9274813	0.76722	0.4278086	0.7404584	0.606293046
Bcl2l1	1	0.1454518	1.0286538	0.177462	1.0539993	0.20118	0.6958344	0.4863722	0.77551644
Ppyr1	1	1.1151651	1.2079485	1.456442	1.0880918	1.08077	0.7115811	0.85638732	0.83349155
Nrg1	1	0.3180806	1.0879564	0.162497	1.0936727	0.11569	0.4625069	0.39078475	0.930069409
Gria1	1	0.1587376	1.0766808	0.159707	1.128912	0.15634	0.2979525	0.07668011	0.481434662
Kcnj3	1	0.1813855	0.9205752	0.232235	1.091633	0.15598	0.4012523	0.23192044	0.074072941
Cdkn1b	1	0.2142488	1.0918104	0.282656	1.0700551	0.21716	0.419078	0.46624523	0.852100482
Vegfa	1	0.1930829	0.9954777	0.151612	1.086648	0.14478	0.9550065	0.26296085	0.19775195
Stat1	1	0.1639588	1.1067083	0.34328	0.9683002	0.10383	0.3722387	0.60712939	0.239840469
Gria3	1	0.1372834	1.0037619	0.133579	1.1200133	0.13098	0.951481	0.05499501	0.072650249
Akt1	1	0.1956789	1.1063846	0.233323	1.0671518	0.1486	0.2816014	0.39069826	0.664015089
Kcnk3	1	0.124703	1.0577111	0.141687	1.1359076	0.16034	0.3454344	0.0420567	0.277960136
Rho	1	0.7055655	1.5924785	0.955966	1.0629357	0.71075	0.1175812	0.84095522	0.172697262
Avpr1a	1	0.4279822	0.9310271	0.512253	1.0166392	0.34879	0.7464172	0.92371704	0.672696138
Adora2a	1	0.456923	1.4819065	1.313241	1.0379457	0.30069	0.1916671	0.45970384	0.311882682
Dnmt1	1	0.1611183	0.9322704	0.116206	1.11357	0.18491	0.3056361	0.14896536	0.021884335
Artn	1	0.3259728	1.0588354	0.19704	0.964254	0.30135	0.6412478	0.79759175	0.435217644
Gfra3	1	0.8370887	0.7040125	0.438846	0.8122191	0.70325	0.516289	0.77835185	0.696533931
Ngfrap1	1	0.0957783	0.8643245	0.201191	1.0765011	0.14556	0.0623991	0.1672422	0.016687252
Kcnk9	1	0.1893251	0.9444416	0.200436	1.1052408	0.16385	0.5327532	0.19120451	0.071389741
Slc17a6	1	0.2403246	1.0663479	0.220951	1.1027335	0.23926	0.532458	0.33920213	0.735749072
Hdac3	1	0.1359754	0.9753336	0.112504	1.0348066	0.11678	0.668549	0.53873261	0.275141681
Rgs2	1	0.1276317	0.9948106	0.175055	1.0418892	0.14209	0.9397222	0.48518497	0.526508165
Prmt3	1	0.1050196	1.0331002	0.094435	1.0853474	0.11882	0.472931	0.09666124	0.307257189
Calcr	1	0.7484225	1.8026439	2.443474	1.1426026	1.10006	0.3821923	0.57295024	0.597089851
Il2	1	0.8267208	0.6942255	0.639293	0.4601241	0.50872	0.5848892	0.15343827	0.330975693
Ccl4	1	0.6681906	1.1187392	0.865452	0.8535996	0.51667	0.7329083	0.58380811	0.422904059
Slc17a7	1	0.1646152	1.1151282	0.255273	1.0872062	0.1826	0.23788	0.26391101	0.785524726
Lpar1	1	0.5168991	0.9143795	0.474031	1.0195623	0.64371	0.7067257	0.93932234	0.693063672
Il1r2	1	0.706421	0.9270217	0.512143	0.6287631	0.37307	0.7987771	0.15467236	0.161955464

Mt3	1	0.1399608	0.9728875	0.196942	0.9637893	0.08288	0.7232472	0.48535664	0.895098931
Il10ra	1	0.2489737	1.2191561	0.479379	0.9580695	0.26309	0.2035759	0.71165274	0.153672272
Cdkn1a	1	0.3138065	0.8237344	0.29664	1.0062149	0.36941	0.2166754	0.96719006	0.255004593
Crebbp	1	0.108347	0.9974262	0.058343	1.0513509	0.11834	0.9497667	0.31216214	0.233255602
Adcyap1r1	1	0.1144418	1.0061184	0.104616	1.0962317	0.13766	0.9030438	0.09654894	0.129880529
Cx3cr1	1	0.3331756	1.0213234	0.171198	1.0829311	0.24174	0.8641295	0.52547906	0.534524671
Agt	1	0.314283	1.0999465	0.66713	1.0231873	0.23171	0.6634916	0.85076223	0.736144815
Crhbp	1	0.0800683	1.0812549	0.201077	1.0611611	0.14756	0.2337963	0.24624934	0.805440665
Fas	1	0.295134	1.0921232	0.288283	0.9149789	0.33563	0.4918645	0.54403982	0.236578464
Kcnh8	1	0.6350173	0.935536	0.653007	1.0068349	0.533	0.4804506	0.49535173	0.915309761
Socs1	1	0.3468381	1.4352003	0.463566	1.3144368	0.42673	0.0272505	0.07832303	0.562052178
Slc17a8	1	0.2559737	0.8835821	0.388069	1.1170824	0.42883	0.4309023	0.45165968	0.232165639
Ccnd1	1	0.1588234	0.9640288	0.352948	0.9552775	0.21838	0.7647357	0.59515864	0.948330055
Gcgr	1	0.7540585	0.9686544	0.761334	1.5995251	1.16975	0.9276439	0.17478913	0.186984773
Cdk2	1	0.2206847	1.0269344	0.321644	0.9346714	0.18902	0.8270141	0.47741378	0.450524387
Ehmt2	1	0.1997737	0.971679	0.133292	1.063572	0.15917	0.7205565	0.43311149	0.193028016
Hdac10	1	0.1826674	0.9305748	0.13996	1.0186883	0.18996	0.3617574	0.82073233	0.270283098
Zfp91	1	0.1871816	0.9716131	0.131784	1.0696579	0.17691	0.706161	0.39298053	0.192651404
Hdac5	1	0.1966763	1.0664634	0.356775	0.9806333	0.08907	0.6030954	0.77841158	0.470683672
Col1a1	1	0.4632006	1.0110513	0.436967	0.9251089	0.34846	0.9571921	0.68266541	0.639835869
Ncoa3	1	0.2393322	0.9535167	0.220143	1.0922848	0.25035	0.6597113	0.39866166	0.218975877
Elk4	1	0.1589437	0.9664688	0.171141	1.060868	0.16885	0.655556	0.4053988	0.243195914
S1pr3	1	0.1871077	1.030923	0.132047	1.1946588	0.11123	0.6467913	0.0208802	0.033973347
Ngf	1	0.1369044	0.8858645	0.282187	1.0806309	0.20154	0.2508435	0.29291965	0.098942558
Prmt6	1	0.2280245	1.0794396	0.1747	1.1617923	0.29333	0.4022685	0.17217173	0.47398851
Sirt1	1	0.1399857	0.9313797	0.107649	1.003787	0.1516	0.2435924	0.95315354	0.251471941
Hdac6	1	0.1373015	1.0263114	0.219113	1.0473497	0.11975	0.7465367	0.41232681	0.795295183
Ncoa1	1	0.1088615	0.9434684	0.061896	1.0555216	0.05984	0.18398	0.16991312	0.000907532
Pik3cg	1	0.2295337	1.1359883	0.232741	1.0856086	0.28725	0.2066816	0.45775406	0.681974181
Hdac9	1	0.196469	1.0177831	0.142593	1.0746967	0.12848	0.8232745	0.32090302	0.372742371
Cxxc1	1	0.1192624	0.9277779	0.091709	1.0036112	0.08105	0.1537079	0.93685112	0.072606128
Hdac11	1	0.112186	1.1576649	0.201696	1.0963659	0.16735	0.0398708	0.13436464	0.479010814
Gcn5l2	1	0.1343172	0.976869	0.087725	1.0616049	0.16923	0.6624337	0.36481364	0.196092272
Dusp14	1	0.1112585	0.9592296	0.151188	1.0919622	0.16281	0.4959495	0.14395852	0.084097534
Tgfb1i1	1	0.1804422	0.9847443	0.189943	1.1141585	0.14048	0.85627	0.12488842	0.107207216
Hdac2	1	0.0703517	0.9208553	0.116311	0.9662567	0.10379	0.0762313	0.39002491	0.38097207
Ehmt1	1	0.2032749	0.9577503	0.141609	1.0575279	0.13962	0.6049294	0.46360329	0.140761928
Ncoa6	1	0.1239095	0.8927346	0.148839	1.0223525	0.11654	0.0953	0.67587251	0.048499118
Ccne2	1	0.2120912	0.9113085	0.180395	1.026212	0.15604	0.3336645	0.75267375	0.154783853
Bai1	1	0.2368495	1.0164065	0.257013	1.1076906	0.19243	0.8836974	0.27004868	0.38985224
Grm2	1	0.253431	0.8114751	0.235631	0.890178	0.2729	0.104962	0.35092594	0.512471466
Hdac4	1	0.2163628	0.9665558	0.162642	1.0194638	0.10286	0.7062779	0.79864587	0.403305726
Hdac8	1	0.1994628	1.0447005	0.181521	1.0516792	0.11402	0.6102182	0.48118479	0.920333355
Prkca	1	0.1286405	1.0318766	0.148791	1.0639425	0.16512	0.6134585	0.3321454	0.663563564
Prmt5	1	0.0902356	0.9844632	0.108809	1.0080849	0.06964	0.7308155	0.82203259	0.576226477
Nrg2	1	0.2709301	0.9753421	0.167231	1.1131204	0.16978	0.8146995	0.27163133	0.093174933
Hdac7	1	0.2038487	1.1143368	0.322366	1.1268895	0.16323	0.346594	0.13439172	0.914605942

Ptgdr	1	0.352226	1.0033443	0.541532	1.5398501	0.6441	0.9868845	0.02599635	0.067543035
Lpar2	1	0.3759095	1.130793	0.40191	0.9107688	0.42271	0.4625888	0.61445346	0.262341548
Ep300	1	0.164317	0.9571697	0.196791	1.0538808	0.18832	0.6019508	0.49223251	0.289096904
Rplp1	1	0.0811373	0.9614848	0.088761	0.9750339	0.06491	0.3245913	0.44889858	0.706706631
Hprt1	1	0.1285605	1.0181714	0.120405	1.0279799	0.09978	0.7501003	0.58678862	0.848381584
Actb	1	0.1074953	1.0171289	0.069305	0.9919517	0.0956	0.6851754	0.85864829	0.524144637

Supplementary Table 2: Human subject demographics (n).

Demographic	Combat-exposed without PTSD	PTSD
Gender	Male (3)	Male (5), Female (1)
Ethnicity	Non-Hispanic (3)	Hispanic (1), Non-Hispanic (5)
Mean age ± SEM	36.33 ± 5.33	34.167 ± 2.58
Race	Caucasian (2), African American (1)	Caucaisian (3), African American (3)
Education	1-3 years of college (2), completed college (1)	1-3 years of college (5), completed college (1)
Marital status	Never married (2), married (1)	Never married (1), married (4), divorced (1)
Employment	Full-time (2), consultant (1)	Part-time (2), retired (1), long-term disability (3)
Service	Army (2), Reservist (1)	Army (4), Navy (1), Marines (1)
Active duty	Yes (1), no (2)	No (6)
Service Connection	Yes (1), no (2)	Yes (6)

Supplementary Table 3: Results of the repeated measures ANOVA analysis of each of the assessments including model selection.

Name Sort Order	**Variable tested**	**RMA Unadj pvalue best model fit**	**Demographic Covariates Model Best Fit**	**AIC, model with no demographic covariates**	**AIC, Race alone model**	**AIC, Age alone model**	**AIC, Age + Race model**	**DELTA AIC: Age Alone AIC - best fit AIC (highlight delta AIC >2)**	**RMA Unadj p value age model fit**	**Q value positive false discovery rate age model fit**
1	NFQ_1	0.011	No covariates	60.3165385	60.69	61.53	61.1404	1.21581866	0.00602	0.012274
2	NFQ_2	0.0126	No covariates	60.5171523	61.14	61.64	61.9191	1.120858943	0.00806	0.013624
3	NDQ_1	0.4996	Age alone	63.7804537	65.26	58.92	59.6937	0	0.49962	0.204755
4	NDQ_2	0.3609	Age + Race	65.4389376	67.27	62.12	61.8747	0.249611089	0.53879	0.216434
5	NDQ_3	0.9155	Age alone	63.7656154	65.36	57.35	58.8662	0	0.91547	0.322791
6	NDQ_4	0.2662	Age alone	64.8868369	66.7	63.02	63.8532	0	0.26623	0.124157
7	NDQ_5	0.3231	Age alone	63.4591304	64.65	57.98	59.2693	0	0.32309	0.145653
8	NDQ_6	0.0937	Age alone	63.3201826	64.88	62.95	63.7281	0	0.09367	0.056932
9	NDQ_7	0.1045	Age + Race	62.7469336	64.41	59.9	58.2699	1.625447352	0.13895	0.078301
10	NDQ_8	0.0213	No covariates	61.7905198	63.57	63.36	64.7373	1.571855615	0.02626	0.030972
11	NDQ_9	0.0501	Race alone	64.9250081	62.95	64.35	64.4216	1.397692604	0.01594	0.022303
12	NDQ_10	0.0312	Age + Race	66.4810999	65.93	64.12	62.86	1.259410579	0.07316	0.052072
13	NDQ_11	0.0002	Race alone	61.0556894	60.07	62.65	61.9037	2.576692852	0.00248	0.007732

14	NDQ_12	0.5992	Age alone	67.2122764	69.14	63.85	65.3314	0	0.59916	0.229921
15	NDQ_13	0.2646	Age alone	65.9260214	67.91	64.07	65.3829	0	0.26465	0.124157
16	ISI_1a	0.1833	Age alone	64.8213756	66.64	63.11	64.2714	0	0.18331	0.094172
17	ISI_2	0.2199	Age alone	65.6083879	67.42	64.09	65.1596	0	0.21993	0.110161
18	ISI_3	0.9296	Age alone	67.4678148	69.18	64.25	64.9751	0	0.92957	0.322791
19	ISI_4	0.3297	Age + Race	66.8400953	68.77	64.12	63.9796	0.14418813	0.58651	0.22881
20	ISI_5	0.1684	Age alone	65.5432055	67.44	63.95	65.6417	0	0.16843	0.089917
21	ISI_total	0.0457	Age alone	63.3158688	65.11	61.63	63.6124	0	0.04565	0.042357
22	PCLM_1	0.6007	Age alone	64.9121569	66.52	60.78	61.3825	0	0.60069	0.229921
23	PCLM_2	0.0696	Age alone	62.776994	63.72	62.1	62.8313	0	0.06962	0.052072
24	PCLM_3	0.1222	Age alone	66.4230679	66.72	64.58	64.9951	0	0.12216	0.070806
25	PCLM_4	0.5807	Age alone	64.9809491	66.34	60.16	61.5016	0	0.58073	0.228753
26	PCLM_6	0.0342	No covariates	62.0019946	63.5	62.87	63.9215	0.86417326	0.03523	0.036647
27	PCLM_7	0.0611	Age alone	63.0758028	64.22	63.07	63.9437	0	0.06109	0.049951
28	PCLM_9	0.04	Race alone	63.7214948	62.59	64.09	63.9568	1.497915901	0.01368	0.019822
29	PCLM_10	0.0131	Race alone	63.1615101	61.73	64.19	63.6775	2.458268769	0.00772	0.01362
30	PCLM_11	0.0034	Race alone	61.9407729	60.92	63.69	62.889	2.773997125	0.00491	0.011059
31	PCLM_12	0.0065	Race alone	62.4895376	62.42	62.99	62.8518	0.568322531	0.00923	0.014987
32	PCLM_13	0.0666	No covariates	63.8764848	65.87	64.14	65.1117	0.264375643	0.08858	0.056155
33	PCLM_14	0.8213	Age alone	66.8414582	68.44	61.71	63.5794	0	0.82131	0.297521
34	PCLM_15	0.0029	No covariates	59.2118509	61.17	61.21	63.1661	1.998584413	0.00434	0.010362
35	PCLM_16	0.0735	No covariates	64.2331635	66.17	64.51	64.8666	0.276020721	0.09402	0.056932
36	PCLM_17	0.0354	No covariates	63.2746738	65.07	64.03	65.3375	0.750593895	0.04287	0.041413
37	PCLM_Total	0.0186	No covariates	61.3055444	61.7	63.29	63.6784	1.989430528	0.01021	0.01594
38	CES_1	0.8358	Age alone	67.86521	69.47	64.6	65.2108	0	0.83578	0.300086
39	CES_2	0.064	Age alone	61.9226447	63.55	57.82	58.6138	0	0.06402	0.049951
40	CES_3	0.5608	Age alone	67.5631545	69.26	64.46	65.4803	0	0.56078	0.223061
41	CES_4	0.0814	Age alone	63.8832832	65.78	63.68	64.9897	0	0.08136	0.054531
42	CES_5	0.0076	Age + Race	61.6926809	61.28	60.12	58.7713	1.349597869	0.00628	0.012274
43	CES_6	0.0224	Age alone	59.330069	61.21	57.35	58.716	0	0.02238	0.028371
44	CES_7	0.8178	Age alone	66.8086913	68.39	62.87	64.0732	0	0.81776	0.297521
45	CES_Total	0.0863	Age alone	65.3899936	66.11	63.7	63.8808	0	0.08629	0.056155
46	BDI_1	0.2437	Age alone	66.20466	66.93	63.35	64.3546	0	0.24365	0.117687
47	BDI_2	0.2428	Age alone	64.6726509	65.19	60.71	62.1088	0	0.2428	0.117687
48	BDI_3	0.0883	Age alone	65.1672718	64.98	63.19	63.8838	0	0.08831	0.056155
49	BDI_4	0.0776	Age alone	64.8818834	64.26	61.86	63.4346	0	0.07761	0.05337
50	BDI_6	0.2317	Age alone	60.0655647	61.98	48.28	49.8839	0	0.23171	0.114645
51	BDI_7	0.1244	Age alone	65.6011883	66.28	63.66	63.9245	0	0.12445	0.071115
52	BDI_8	0.0713	Age alone	63.137041	63.27	59.29	61.2234	0	0.07128	0.052072
53	BDI_9	0.2999	Age alone	66.6730757	67.82	64.01	64.5621	0	0.29993	0.136991
54	BDI_10	0.0314	Age + Race	66.3450473	66.22	64.43	64.2822	0.14287832	0.09112	0.056876
55	BDI_11	0.3707	Age alone	66.7321662	67.74	60.93	62.8789	0	0.37074	0.163498
56	BDI_12	0.1426	Age alone	64.8321916	65.14	61.34	62.9491	0	0.14261	0.079263
57	BDI_13	0.0019	Race alone	59.2637962	56.41	61.11	58.4107	4.695806539	0.00019	0.001879
58	BDI_14	0.0392	Age alone	64.5743483	64.81	63.78	63.7986	0	0.03916	0.038753
59	BDI_15	0.0763	Age alone	64.1368213	65.89	63.64	64.8502	0	0.0763	0.05337
60	BDI_16	0.0045	No covariates	58.951984	60.75	60.61	62.5862	1.659458004	0.00394	0.010362
61	BDI_17	0.9308	Age alone	66.8940496	68.53	60.59	62.1215	0	0.93084	0.322791
62	BDI_18	0.0341	Race alone	65.2253673	63.53	63.66	64.2242	0.122641318	0.03002	0.03205
63	BDI_19	0.0558	Age alone	65.2577592	65.5	64.57	64.9528	0	0.05576	0.04918
64	BDI_20	0.5021	No covariates	57.7755724	59.53	59.54	61.4148	1.763987115	0.00153	0.00581
65	BDI_21	0.8062	Age alone	67.6187162	69.2	64.11	64.1602	0	0.80616	0.297521
66	BDI_Total	0.022	Race alone	63.468428	62.78	62.99	63.671	0.214053684	0.02735	0.030972
67	PSQL_3_vs_PSQL_1	0.2559	Age alone	61.021633	63.02	52.82	54.8092	0	0.25592	0.122158
68	PSQI_5a	0.9626	Age alone	65.1909655	66.81	61.12	61.5114	0	0.96255	0.328178
69	PSQI_5b	0.0017	Age alone	55.4806145	56.75	51.21	53.073	0	0.00172	0.00581
70	PSQI_5c	0.6542	Age alone	67.6239403	69.42	64.56	65.6948	0	0.65418	0.248051
71	PSQI_5d	0.0759	Age + Race	65.5301108	65.38	63.61	62.9951	0.616414978	0.06263	0.049951
72	PSQI_5e	0.9049	Age alone	64.0276086	65.68	58.75	60.3859	0	0.90492	0.322059
73	PSQI_5f	0.9402	Age alone	64.9500825	66.57	59.79	61.1415	0	0.94022	0.323279
74	PSQI_5g	0.3897	Age alone	67.5820403	68.78	64.83	65.6803	0	0.38968	0.168194
75	PSQI_6	0.759	Age alone	66.3847044	67.99	62.7	63.5933	0	0.75903	0.285148
76	PSQI_7a	0.042	Age + Race	66.5306123	66.15	63.88	62.3129	1.566232142	0.08199	0.054531
77	PSQI_7c	0.1857	Age alone	66.6359309	67.32	64.4	65.4109	0	0.18569	0.094172
78	PSQI_8	0.5293	Age alone	64.7290523	66.05	60.22	61.4577	0	0.52927	0.214737

79	PSQI_9	0.1554	Age alone	66.3765915	66.61	63.96	64.711	0	0.15545	0.084091
80	PSQI_10	0.0077	Age alone	62.0099086	63.87	52.95	54.5701	0	0.47288	0.195775
81	PSQIa_12a	0.1849	Age alone	66.8764837	67.61	64.47	64.6022	0	0.18486	0.094172
82	PSQIa_12b	0.0278	Race alone	62.1673641	61.46	62.62	62.0133	1.16772448	0.00635	0.012274
83	PSQIa_12c	0.3173	No covariates	61.3950351	63.02	62.92	64.0688	1.527904147	0.02824	0.030972
84	PSQIa_12d	0.0617	Age alone	64.0900391	64.16	62.45	63.792	0	0.06171	0.049951
85	PSQIa_12e	0.3005	Age alone	65.56708	67.5	63.77	64.3962	0	0.3005	0.136991
86	PSQIa_12f	0.0453	Age + Race	65.6712961	65.7	64.22	64.1227	0.096304466	0.06576	0.050338
87	PSQIa_12g	0.0459	Age alone	64.4962771	65.08	63.78	64.0803	0	0.04593	0.042357
88	PSQIa_13a	0.0388	No covariates	63.2627682	65.26	63.93	65.4511	0.670828436	0.05278	0.04759
89	PSQIa_13b	0.4626	Age alone	65.3265442	66.66	58.71	60.1922	0	0.46258	0.194725
90	Iowa1	0.3895	Age alone	66.8289777	68.77	64.78	65.6878	0	0.38946	0.168194
91	Iowa2	0.1856	Age + Race	67.321805	67.21	64.67	64.0799	0.586944677	0.1503	0.082407
92	Iowa3	0.4655	Age alone	67.3834185	68.76	64.46	65.1908	0	0.46555	0.194725
93	CAPS1	0.0719	Age alone	63.6672158	65.4	63.21	64.3879	0	0.07193	0.052072
94	CAPS2	0.0293	No covariates	62.2414764	63.61	63.02	64.0205	0.778404135	0.02817	0.030972
95	CAPS3	0.1837	Age alone	65.9917998	67.42	64.55	65.48	0	0.18374	0.094172
96	CAPS4	0.0367	Age alone	62.7641297	64.68	60.84	62.3246	0	0.03673	0.037252
97	CAPS5	0.0578	No covariates	64.0894486	65.73	64.59	65.6816	0.496145038	0.06319	0.049951
98	CAPS6	0.0089	No covariates	60.7251713	61.75	62.08	63.3182	1.358386333	0.0072	0.013286
99	CAPS7	0.0131	Age alone	59.0839947	61.05	58.81	60.4485	0	0.01312	0.019717
100	CAPS8	0.0029	Race alone	60.5372274	57.88	60.66	57.9155	2.788230347	0.00056	0.002936
101	CAPS9	0.0003	Race alone	56.3663243	55.04	58.11	56.773	3.076306841	#######	0.001879
102	CAPS10	0.0039	Race alone	60.959799	60.85	62.96	62.7497	2.103418111	0.00402	0.010362
103	CAPS11	0.0002	No covariates	55.1035677	56.8	57.03	58.7818	1.928179392	0.00028	0.001879
104	CAPS12	0.0378	Age + Race	66.2378419	66.27	64.26	64.1856	0.075593735	0.10063	0.060039
105	CAPS13	0.0006	Age alone	54.6949688	56.66	53.47	55.1149	0	0.00058	0.002936
106	CAPS14	0.0219	No covariates	61.6608627	63.46	62.38	63.7405	0.717668971	0.02722	0.030972
107	CAPS15	0.0202	No covariates	62.309163	63.82	63.58	64.7565	1.267156022	0.02115	0.027687
108	CAPS16	0.0547	No covariates	63.8320445	65.53	64.19	65.3474	0.360787887	0.06194	0.049951
109	CAPS17	0.0002	No covariates	54.6754614	55.88	56.58	57.6688	1.908673265	0.00018	0.001879
110	CAPS_total	0.0003	No covariates	55.7920052	56.75	57.17	58.3372	1.376743529	0.00024	0.001879
111	CAPS_ reexperiencing	0.0035	No covariates	59.362525	60.98	61.34	62.9784	1.975347823	0.00414	0.010362
112	CAPS_ avoidance	0.0003	Race alone	56.0400937	55.76	57.53	57.2051	1.775110376	0.00012	0.001879
113	CAPS_arousal	0.0005	No covariates	56.5887959	58.32	58.53	60.1707	1.94182208	0.00073	0.003269
114	MDD	0.1086	Age alone	65.6479198	66.69	63.91	64.3752	0	0.10861	0.063864
115	GAD	0.441	Age alone	64.9038182	66.82	56.83	58.6465	0	0.441	0.188343
116	Insomnia	0.0017	Age alone	56.0594259	58.05	53.36	55.2158	0	0.00169	0.00581
117	Sleep Apnea	0.8104	Age alone	67.1285362	68.8	62.45	63.3926	0	0.81041	0.297521
118	RBD	0.3482	Age alone	67.4330799	68.57	64.73	65.3582	0	0.34823	0.155258
