# Supplementary material for: Sphingosine-1-phosphate receptor 3 in the medial prefrontal cortex promotes stress resilience by reducing inflammatory processes
Source: Nat Commun. 2019 Jul 17;10:3146. doi: 10.1038/s41467-019-10904-8 (PMC6637233; doi:10.1038/s41467-019-10904-8)
Supplement: Supplementary file 2 — Reporting Summary [file 41467_2019_10904_MOESM2_ESM.pdf]

nature research

Corresponding author(s): *Double-blind peer review submissions: write DBPR and your manuscript number here instead of author names.*

Reporting Summary

Nature Research wishes to improve the reproducibility of the work that we publish. This form provides structure for consistency and transparency in reporting. For further information on Nature Research policies, see Authors & Referees and the Editorial Policy Checklist.

Statistical parameters

When statistical analyses are reported, confirm that the following items are present in the relevant location (e.g. figure legend, table legend, main text, or Methods section).

n/a	Confirmed	
☐	☒	The exact sample size (*n*) for each experimental group/condition, given as a discrete number and unit of measurement
☐	☒	An indication of whether measurements were taken from distinct samples or whether the same sample was measured repeatedly
☐	☒	The statistical test(s) used AND whether they are one- or two-sided<br>*Only common tests should be described solely by name; describe more complex techniques in the Methods section.*
☐	☒	A description of all covariates tested
☐	☒	A description of any assumptions or corrections, such as tests of normality and adjustment for multiple comparisons
☐	☒	A full description of the statistics including central tendency (e.g. means) or other basic estimates (e.g. regression coefficient) AND variation (e.g. standard deviation) or associated estimates of uncertainty (e.g. confidence intervals)
☐	☒	For null hypothesis testing, the test statistic (e.g. *F*, *t*, *r*) with confidence intervals, effect sizes, degrees of freedom and *P* value noted<br>*Give P values as exact values whenever suitable.*
☒	☐	For Bayesian analysis, information on the choice of priors and Markov chain Monte Carlo settings
☒	☐	For hierarchical and complex designs, identification of the appropriate level for tests and full reporting of outcomes
☐	☒	Estimates of effect sizes (e.g. Cohen's *d*, Pearson's *r*), indicating how they were calculated
☐	☒	Clearly defined error bars<br>*State explicitly what error bars represent (e.g. SD, SE, CI)*

Our web collection on statistics for biologists may be useful.

Software and code

Policy information about availability of computer code

Data collection	n/a
Data analysis	Detailed description of code used for statistical analyses used for calculating correlations and adjusted p-values for covariates from human subjects is reported in the Methods section

For manuscripts utilizing custom algorithms or software that are central to the research but not yet described in published literature, software must be made available to editors/reviewers upon request. We strongly encourage code deposition in a community repository (e.g. GitHub). See the Nature Research guidelines for submitting code & software for further information.

Data

Policy information about availability of data

All manuscripts must include a data availability statement. This statement should provide the following information, where applicable:

- Accession codes, unique identifiers, or web links for publicly available datasets
- A list of figures that have associated raw data
- A description of any restrictions on data availability

The data from each figure that support the findings of this study are available from the corresponding author upon request.

Field-specific reporting

Please select the best fit for your research. If you are not sure, read the appropriate sections before making your selection.

☒ Life sciences ☐ Behavioural & social sciences ☐ Ecological, evolutionary & environmental sciences

For a reference copy of the document with all sections, see nature.com/authors/policies/ReportingSummary-flat.pdf

Life sciences study design

All studies must disclose on these points even when the disclosure is negative.

Sample size	In line with similar experiments performed in our laboratory, a power analysis assuming a medium effect size was used to determine than an n of 6-9 would be sufficient to determine significance.
Data exclusions	Data varying three standard deviations from the group mean were excluded from analysis. These exclusions were rare.
Replication	In Figure 1, increased S1PR3 expression in resilient rats as assessed by mRNA levels was supported by increased S1PR3 protein expression. One of the main findings of this paper, S1PR3 knockdown causing behavioral and neuroendocrine phenotypes similar to stress-vulnerable rats, was initially reported in Figure 2 and reproduced in Figure 3 when S1PR3 knock-down rats were compared to control rats and rats that had a cytokine knocked down or were administered infliximab. All experiments in which infliximab rescued behavioral effects were replicated by TNFa knockdown. Each key phenotype or molecular change reported in S1PR3 knock-down rats was reported to be the opposite in S1PR3 over-expressing rats. Additionally, compared to combat-exposed non-PTSD veterans, S1PR3 expression in PTSD patients was reduced at three discrete time points. In these subjects, mean S1PR3 expression inversely correlated with PTSD symptom severity as assessed by two different, independent assessments of PTSD, multiple symptom subclusters, and depression symptoms.
Randomization	Singly housed rats were numbered randomly upon entry to the animal facility and placed in groups by our lab manager or animal care staff, who was not directly involved in data collection or analysis, prior to the begin of each study
Blinding	Investigators were blinded to group allocation during data collection and analysis

Reporting for specific materials, systems and methods

Materials & experimental systems

n/a	Involved in the study
☐	☒ Unique biological materials
☐	☒ Antibodies
☒	☐ Eukaryotic cell lines
☒	☐ Palaeontology
☐	☒ Animals and other organisms
☐	☒ Human research participants

Methods

n/a	Involved in the study
☒	☐ ChIP-seq
☒	☐ Flow cytometry
☒	☐ MRI-based neuroimaging

Unique biological materials

Policy information about availability of materials

Obtaining unique materials	All materials used in these experiments are available through commercial vendors. AAV1-S1PR3-GFP and AAV1-GFP are available through the Penn Vector Core.

Antibodies

Antibodies used	rabbit anti-S1PR3/Edg3 (bs-7541R, 1:100, BIOSS), guinea pig anti-NeuN (ABN90, 1:1000, EMD Millipore), mouse anti-GAD67 (MAB5406, 1:5000, EMD Millipore), goat anti-GFP (ab5450, Abcam, 1:2000), rabbit anti-IBA1 (019-19741, Wako, 1:250), rabbit anti-TNFa (NBP1-19532, Novus Biologicals, 1:100), rabbit anti-IL1B (sc-7884, 1:100, Santa Cruz), and mouse anti-glucocorticoid receptor (ab9568, Abcam, 1:100)
Validation	All antibodies were confirmed to specifically detect the target antigen using immunohistochemistry by the manufacturer as described on their web site with at least one peer-reviewed publication cited. Additionally, antibodies for S1PR3, TNFa, IL1B, and GR were validated by reduced immunoreactivity following knock-down and are quantified in this manuscript.

Animals and other organisms

Policy information about studies involving animals; ARRIVE guidelines recommended for reporting animal research

Laboratory animals	Male Sprague-Dawley rats, between 2 and 4 months of age, were used in these experiments. Male Long-Evans rats, aged 8-12 months, were used as resident rats.
Wild animals	n/a
Field-collected samples	n/a

Human research participants

Policy information about studies involving human research participants

Population characteristics	Population characteristics for human participants are outlined in Supplementary Table 3.
Recruitment	All human subjects recruited for this study were seeking care at the Corporal Michael J. Crescenz Veteran's Affairs Medical Center in Philadelphia, PA and volunteered to be participants in the study.
